# Accurate Prediction of Inducible Transcription Factor Binding Intensities In Vivo

**DOI:** 10.1371/journal.pgen.1002610

**Published:** 2012-03-29

**Authors:** Michael J. Guertin, André L. Martins, Adam Siepel, John T. Lis

**Affiliations:** 1 Department of Molecular Biology and Genetics, Cornell University, Ithaca, New York, United States of America; 2 Department of Biological Statistics and Computational Biology, Cornell University, Ithaca, New York, United States of America; Stanford University School of Medicine, United States of America

## Abstract

DNA sequence and local chromatin landscape act jointly to determine transcription factor (TF) binding intensity profiles. To disentangle these influences, we developed an experimental approach, called protein/DNA binding followed by high-throughput sequencing (PB–seq), that allows the binding energy landscape to be characterized genome-wide in the absence of chromatin. We applied our methods to the *Drosophila* Heat Shock Factor (HSF), which inducibly binds a target DNA sequence element (HSE) following heat shock stress. PB–seq involves incubating sheared naked genomic DNA with recombinant HSF, partitioning the HSF–bound and HSF–free DNA, and then detecting HSF–bound DNA by high-throughput sequencing. We compared PB–seq binding profiles with ones observed in vivo by ChIP–seq and developed statistical models to predict the observed departures from idealized binding patterns based on covariates describing the local chromatin environment. We found that DNase I hypersensitivity and tetra-acetylation of H4 were the most influential covariates in predicting changes in HSF binding affinity. We also investigated the extent to which DNA accessibility, as measured by digital DNase I footprinting data, could be predicted from MNase–seq data and the ChIP–chip profiles for many histone modifications and TFs, and found GAGA element associated factor (GAF), tetra-acetylation of H4, and H4K16 acetylation to be the most predictive covariates. Lastly, we generated an unbiased model of HSF binding sequences, which revealed distinct biophysical properties of the HSF/HSE interaction and a previously unrecognized substructure within the HSE. These findings provide new insights into the interplay between the genomic sequence and the chromatin landscape in determining transcription factor binding intensity.

## Author Summary

Transcription factors (TFs) bind DNA to modulate levels of gene expression. TF binding sites change throughout development, in response to environmental stimuli, and different tissues have distinct TF binding profiles. The mechanism by which TFs discriminate between binding sites in a context dependent manner is an area of active research, but it is clear that the chromatin environment in which potential binding sites reside strongly influences binding. This study used the Heat Shock TF (HSF) to study the effect chromatin has upon induced HSF binding. We implemented an experimental technique to quantify all potential HSF binding sites in the genome. These data were incorporated into a modeling framework along with chromatin landscape information prior to HSF binding to accurately predict the intensities of inducible HSF binding sites. DNase I hypersensitivity and tetra-acetylation of H4 were the most influential covariates in the model. The binding data enabled the development of a more complete HSF/DNA interaction model, providing insight into the biophysical interaction of HSF trimer subunits and target DNA pentamers.

## Introduction

Binding of transcription factors (TFs) to DNA elements is necessary to establish and maintain functional changes in gene expression levels. The mechanism by which these factors seek out and bind to their cognate motif elements remains an area of active investigation (reviewed in [1]). TFs are present at cellular concentrations that allow binding to sites that are degenerate from the consensus sequences, and genomes of eukaryotes are littered with potential degenerate binding sites; however, only a small fraction of potential binding sites are recognized in vivo. Moreover, TF binding sites vary dependent upon cell type and cellular conditions. In vivo, TF binding is potentially dependent upon motif accessibility and the surrounding chromatin landscape. Therefore, determining a comprehensive set of potential genomic binding sites and quantifying the joint effects of DNA sequence and chromatin landscape upon binding intensity remains a challenge.

Experimental approaches to characterize TF binding sites include assays such as ChIP-seq, protein binding microarrays (PBM) [2], iterative rounds of protein-DNA binding and selection with a complex oligonucleotide library [3], or extrapolation from DNase I hypersensitivity regions [4]. However, perhaps the most direct way to determine all potential TF binding sites within a genome is to incubate purified TF and naked sheared genomic DNA in vitro, and then specifically quantify the TF-bound DNA [5]. This in vitro method allows binding sites to be interrogated in their native sequence context without the confounding effects of chromatin and cooperation between chromatin-bound factors.

It is challenging to predict in vivo TF binding accurately even when all potential in vitro binding sites have been characterized, because the chromatin landscape dramatically influences binding and it changes dynamically with development and with alterations in cellular nutrition and environment [6], [7]. Recent TF binding site modeling efforts have considered genomic nucleosome occupancy or DNase I hypersensitivity data to account for the effect chromatin has upon in vivo TF occupancy [8]–[11]. However, these models are limited in that they rely upon genomic accessibility data and TF binding data produced under the same conditions. To date there are no data sets that describe the full set of potential TF binding sites, the chromatin state data prior to binding, and occupied binding sites in vivo, in a single inducible system. Integration of these three data sets would allow one to decouple the effect TF binding has upon chromatin state from the effect pre-existing chromatin state has upon induced TF binding.

The heat shock response of *Drosophila* is a model system extensively used to study the general functions of sequence specific activators and how they function to regulate transcription (reviewed in [12]). The master regulator of the heat shock genes, Heat Shock Factor (HSF), has a modest affinity for DNA under non-stress conditions [6], [13], [14], and upon stress, HSF homotrimerizes and inducibly binds to a conserved consensus motif at over 400 sites in the *Drosophila* genome [6], [14]. While over 95% of the HSF binding sites contain an underlying HSF sequence motif element (HSE), the vast majority of predicted genomic HSEs remain HSF–free following heat shock. Therefore, the chromatin landscape most likely plays a prominent role in determining binding of HSF.

Here, we describe an experimental technique, protein/DNA binding followed by high-throughput sequencing (PB–seq), to quantify the binding potential of all binding sites within a genome. We then develop a quantitative model that incorporates HSF PB–seq data, together with HSF ChIP-seq in *Drosophila* S2 cells [6] and S2 cell chromatin data, that accurately predicts observed in vivo HSF binding profiles. Moreover, our model allows us to quantify the relative importance of the chromatin features influencing HSF binding intensity. Finally, we develop a sequence model that uses HSF PB–seq data to characterizes the relationship between positions within the HSE and provide biophysical insight into the mechanisms by which HSF interacts with its cognate element.

## Results

### Quantification of the absolute binding affinity of all genomic *Drosophila* HSEs

We performed an in vitro binding experiment with purified HSF (Figure S1) and naked, sheared genomic *Drosophila* DNA, to derive an accurate set of potential HSF binding sites in the *Drosophila* genome. HSF–bound DNA was specifically eluted and detected by high throughput sequencing (Methods). The HSF PB–seq experiment yielded 68% of the sequence tags within peaks. In contrast, typical ChIP-seq protocols are more inefficient and the majority of DNA (60% to >99%) sequenced is uninformative background DNA [15].

Peak calling revealed 3952 HSF–binding peaks (*p*<0.01; 2848 peaks were common to both experimental replicates), which include 60% of the previously identified high-confidence HSF binding peaks in vivo [6]. The naïve expectation is that every in vivo HSF peak should have a corresponding in vitro peak, but it is not surprising to observe an incomplete overlap of in vivo by in vitro peaks, for various reasons. As will be discussed, binding sites detected in vivo but not in vitro tend to be more degenerate and have higher DNase I accessibility. Additionally, in vivo binding sites that are dependent upon cooperative interactions with pre-bound chromatin factors, long range DNA interactions, post-translational modifications of HSF [16], higher-order chromatin structure, or bridging protein interactions [17] will not be detected in the current form of PB–seq.

**Figure 1:**
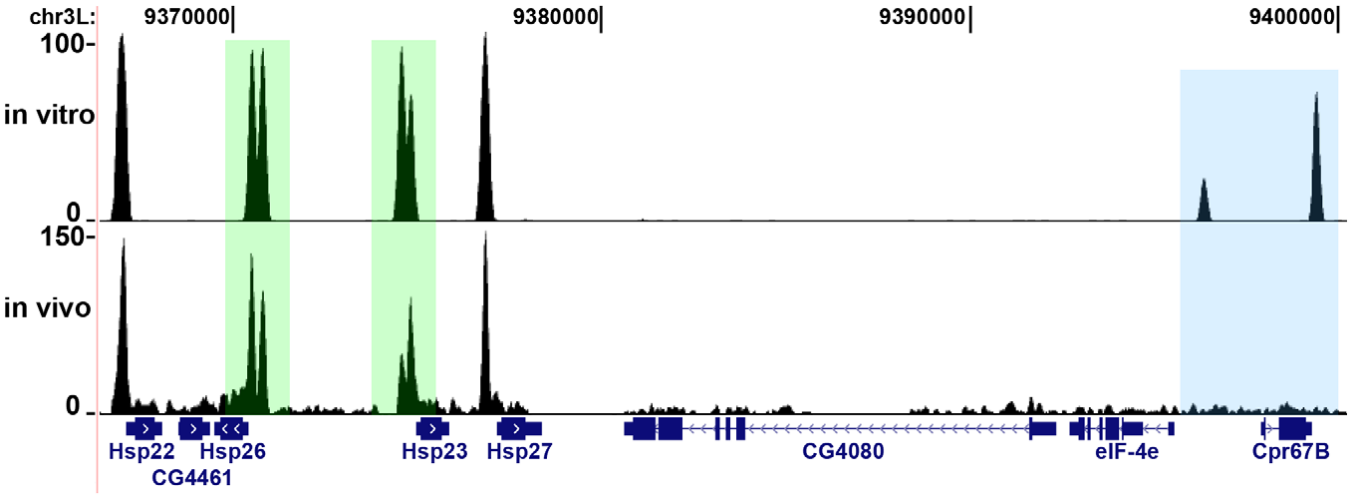
In vitro binding reveals potential HSF binding sites. The blue box highlights strong differences in the usage of potential binding sites in vivo at the Cpr67B locus, while the green boxes highlight differences in the magnitude of binding to major heat shock genes promoters, despite comparable in vitro binding affinities.

Underlying the in vitro binding peaks, we detected 3735 clusters of HSF binding site HSE sequences (2896 in peaks common to both replicates) at 20% HSE False Discovery Rate (FDR). We used clusters of co-occurring sites due to the uncertainty in HSE detection (see Methods). Furthermore, the majority, 3389 clusters (2586 in peaks common to both replicates) are not detectably bound in S2 cells in vivo. Figure 1 shows two examples of in vitro binding sites flanking the Cpr67B gene that are not bound in vivo. Moreover, the in vitro binding data quantifies differences in the in vitro and in vivo HSF binding intensity, such as the peaks within each of the promoters for Hsp23 and Hsp26 (Figure 1).

The PB–seq experiment allows for an estimate of the relative binding intensity of each HSE, based on the number of sequence tags associated with it. To compute the dissociation constant (Kd) values it is necessary to have estimates for both the fraction of bound and free HSE in the PB–seq experiment. Since the PB–seq data only provides information on the bound fraction, we needed to determine the absolute Kds for two HSEs that are found within the PB–seq data in order to provide enough information to estimate the free fraction (see Methods).

**Figure 2:**
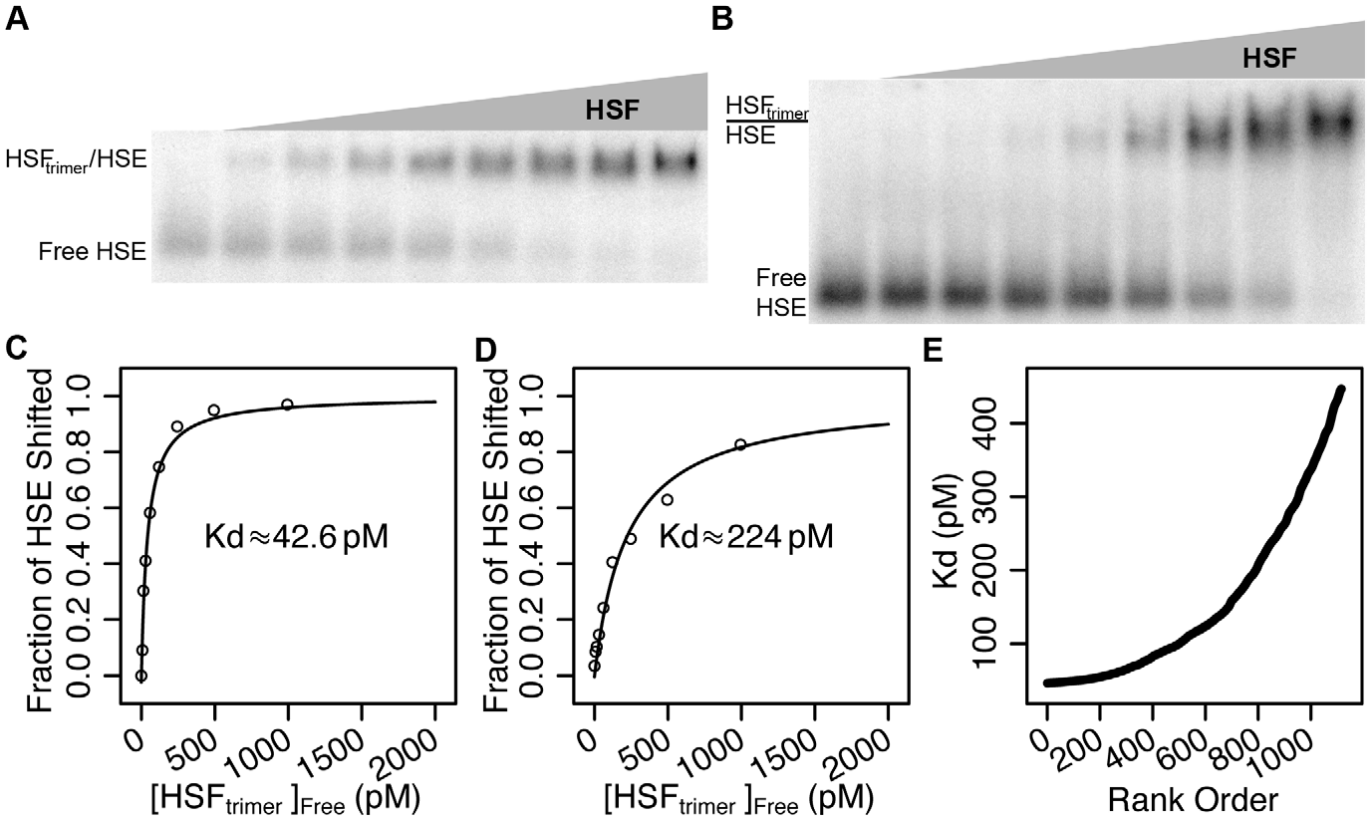
Recombinant HSF binds HSEs with picomolar affinity in vitro. A and B) The mobility of the constant 200 attomole HSE probe shifts into a trimeric-HSF:HSE complex as increasing HSF is added. There is no HSF in the left-most lane, the right-most lane contains 3 nM HSF (1 nM trimeric HSF), and the intervening lanes contain two-fold serial dilutions of HSF. C) A hyperbolic curve based on the Kd equation (see Methods) was modeled using the band shift data, indicating a Kd of 42.6 pM (95% confidence interval of 36.8–49.4 pM). D) A hyperbolic curve based on the Kd equation (see Methods) was modeled using the band shift data, indicating a Kd of 224 pM (95% confidence interval of 181–276 pM). E) The intensity of each isolated HSE in the *Drosophila* genome is transformed to an absolute Kd using the absolute Kds calculated from band shift data in panels A and B. The Kd values range from 40–400 pM.

To generate the HSF/HSE Kd measurements, we performed electrophoretic mobility shift assays (EMSA). The EMSAs were performed with purified HSF and HSEs that are only modestly degenerate from the consensus. We found that HSF binds to the first HSE with ∼42.6 pM interval: 36.8–49.4 pM; Figure 2A and 2C) and the second HSE with ∼224 pM affinity (95% confidence interval: 181–276 pM; Figure 2B and 2D). The resulting two absolute Kd values enabled us to transform PB–seq read depths into absolute Kd values (Figure 2E and Methods). We confirmed the transformation of the relative Kd values to absolute Kds by performing band shifts with genomic HSEs of different predicted Kd values (Figure S2). The experimental verifications of the measurements are within the estimated error of the EMSA confidence interval and the variability between PB–seq replicates (Figure S3).

Taken together, these measurements allow us to characterize the binding energy landscape for HSF across the entire *Drosophila* genome, in the absence of chromatin. Our estimated Kd values for isolated HSEs in the *Drosophila* genome ranged from 40–400 pM (Figure 2E). These in vitro binding results demonstrate the feasibility and efficiency of combining high-throughput detection methods with classic EMSA and competition experiments to quantify the binding energy for the comprehensive set of potential genomic binding sites for a sequence-specific TF.

### Chromatin features and PB–seq data predict HSF binding intensity in vivo

**Figure 3:**
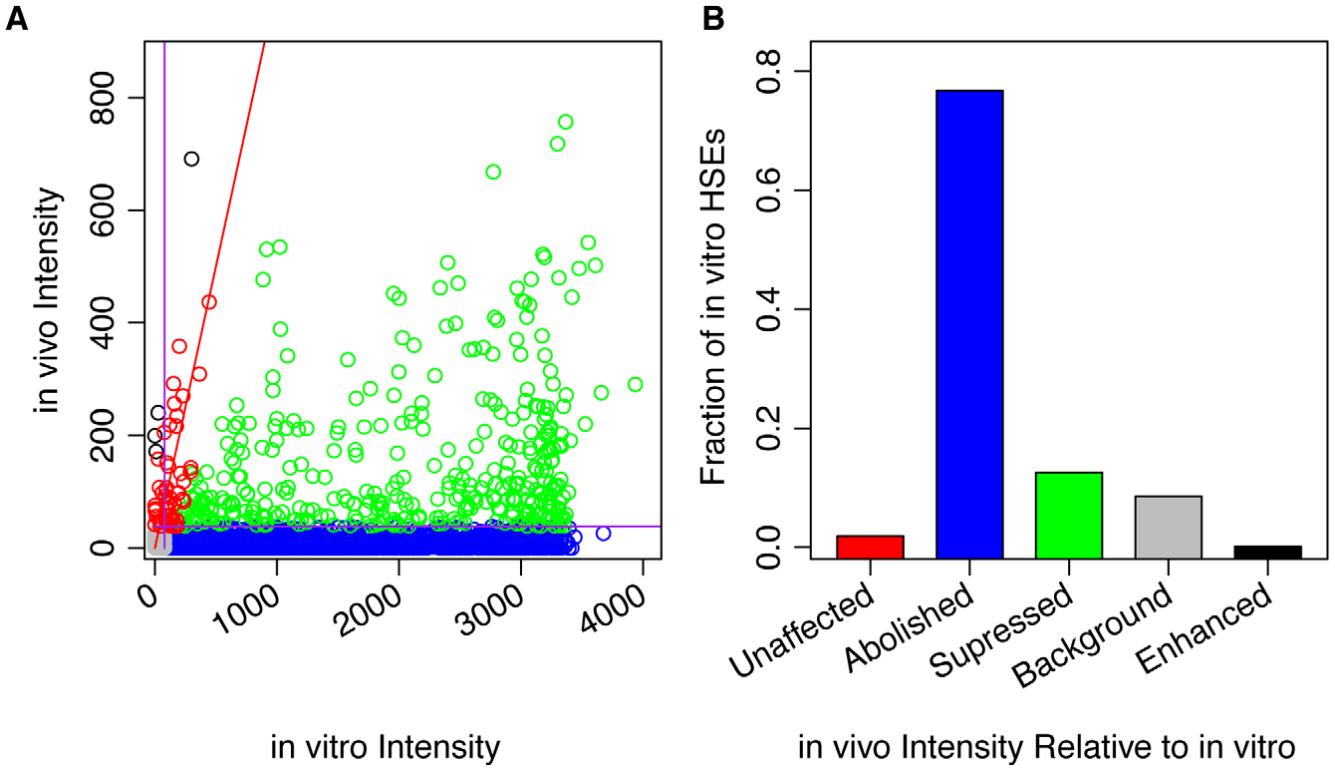
In vitro and in vivo binding of HSF to genomic HSEs do not correlate. A) A scatter plot comparing the observed in vivo HSF binding intensity and in vitro binding intensity for each isolated HSE indicates that the vast majority of in vivo binding is suppressed (green) or abolished (blue), if we assume that the top seven most DNase I hypersensitive isolated HSE clusters provide the best estimates for sites that are minimally influenced by chromatin. After scaling, red points have similar in vivo and in vitro intensity, black points may be enhanced in vivo, while green and blue points are suppressed and abolished, respectively. B) The points from panel A were categorized, and the resulting bar chart shows the relative frequencies of each category.

Our data reveals substantial differences between in vivo and in vitro binding intensities (Figure 3A), underscoring the role of chromatin in determining in vivo binding site selection and affinity. We found DNase I hypersensitivity was the most important predictor of HSF binding; therefore, we scaled the in vivo and the in vitro read counts so that they were approximately equal at in vivo sites with high DNA accessibility (Methods, Figure S4). After this normalization, we partitioned the binding sites that were detectable in vitro into classes: “unaffected” sites, bound at comparable affinities in vivo and in vitro (55 red points in Figure 3A; 2% of all sites); “suppressed” sites, with reduced, but detectable, in vivo intensity (365 green points; 13%); and “abolished” sites, below the in vivo threshold for detection (2223 blue points; 76%). In addition, sites not detectable in vivo or in vitro were labeled “background” (249 gray points; 9%), and sites with stronger relative in vivo intensity compared to in vitro were labeled “enhanced” (4 black points; 0.1%).

**Figure 4:**
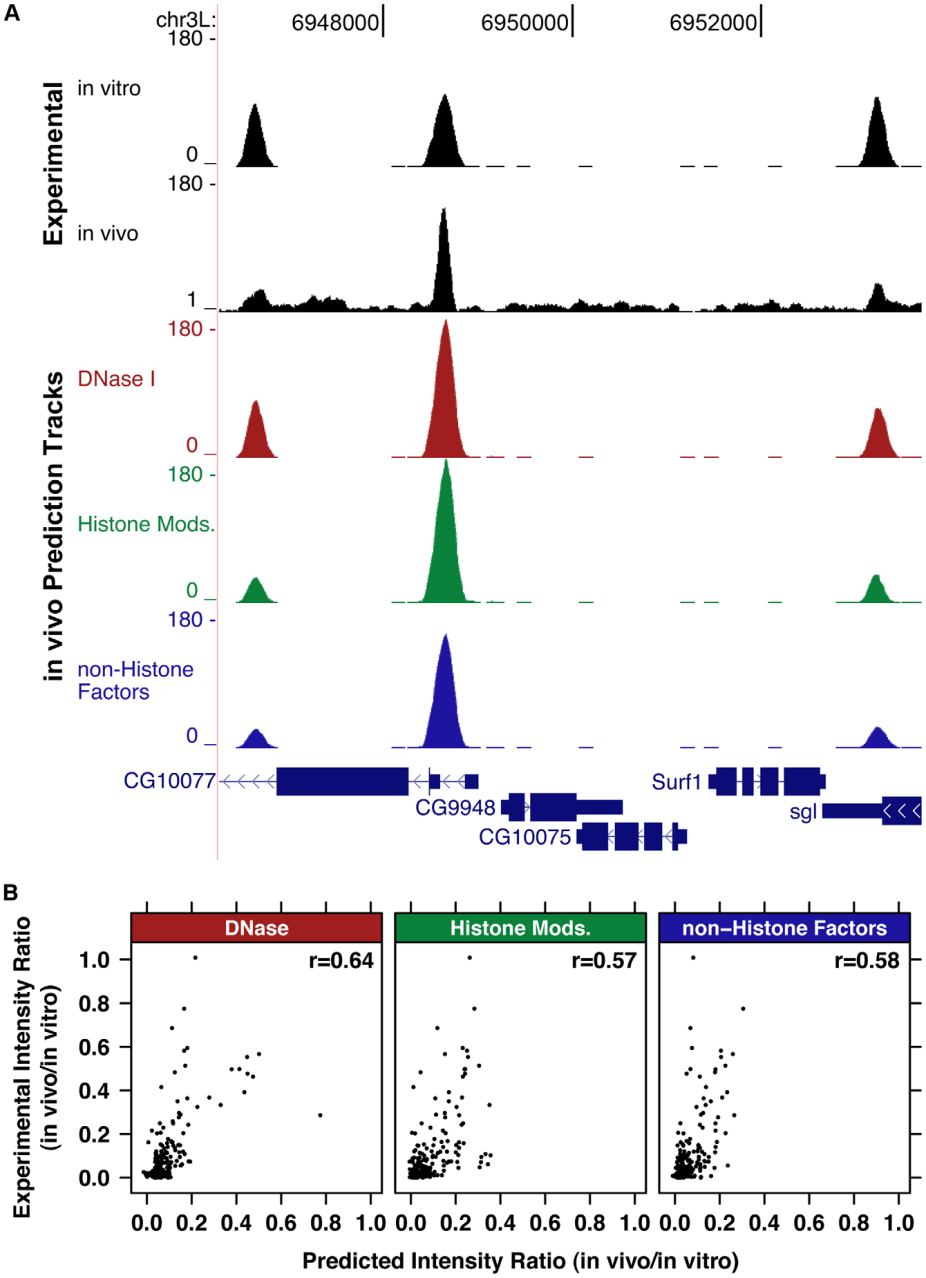
Genomic chromatin and PB–seq data accurately predict in vivo HSF binding intensity. A) The intensity of in vivo ChIP-seq peaks is not recapitulated by in vitro PB–seq data; however, genomic DNase I hypersensitivity data and histone modification ChIP-chip data can be used to accurately predict HSF binding intensity. B) The experimentally determined ratio between in vivo ChIP-seq HSF intensity and in vitro PB–seq intensity is plotted against the predicted in vivo/actual PB–seq ratio. The Pearson correlation for each model is shown.

PB–seq data reveals potential HSF binding sites, providing the opportunity to model the effect that non-stressed chromatin landscape has upon induced HSF binding intensity. There is a wealth of chromatin data available for S2 cells during unstressed conditions [18], [19], and heat-shock induced binding sites of HSF in S2 cells are also known [6]. We used DNase I hypersensitivity data [18], MNase data [19] and ChIP-chip data for 9 factors and 21 histone modifications for unstressed *Drosophila* S2 cells (Table S1) [18] to predict the intensity of inducibly bound in vivo HSF–bound sites (Figure 4A, Figure S5 and Figure S6). For our statistical model, we selected a *rules ensemble* [20], a linear regression model in which some terms are combinations of covariates known as “rules”. This approach allowed us to capture fairly complex interactions between covariates. For example, a rule might apply when H3K27ac and DNase I hypersensitivity both exceeded designated thresholds (value ranges can also be expressed). Each rule's coefficient is added to the predicted value if, and only if, the rule applies. When there is only one covariate, the model reduces to a linear regression.

The Pearson's correlation coefficient between HSF ChIP-seq data for the model incorporating all these data sets was r = 0.62 (Figure S6 and Figure S7). As the large number of covariates brings with it some danger of overfitting, we tested combinations of the four classes of covariates: DNase I hypersensitivity, MNase, histone modifications/variants, and non-histone factors (Figure 4B, Figure S6, Figure S7). Of notice, the correlation of the linear regression model that incorporates DNase I data was r = 0.64 on the test data (Figure 4B and Figure S7B). Our study is consistent with a previous study that obtained r = 0.65 for actual and inferred TF binding intensities using a DNase I dependent model [8].

Other covariate classes produce similar, but lower, correlations. The rules model using histone modifications and histone variants yielded r = 0.57 (Figure 4B and Figure S7), while a rules model incorporating non-histone bound chromatin factors yielded r = 0.58 (Figure 4B and Figure S7). Combining covariate classes further improves the correlation to as much as r = 0.70 (Figure S6 and Figure S7). We also examined the Receiver Operator Curves (ROC) for the different covariate combinations (Figure S8) and found concordant results. If we assume that the PB–seq, genomic ChIP, DNase I-seq, and MNase-seq experiments are maximally resolved and sensitive, with no experimental noise, an approximate upper bound is given by r = 0.90, as observed for two HSF–ChIP-seq replicates [6]. Notably, the higher resolution of the DNase I-seq data, compared to the ChIP-chip data, may be why DNase I-seq alone is strongly predictive in the linear regression model and most influential in the rules ensemble models. Notably, we used the chromatin landscape prior to induced TF binding to predict binding intensity, whereas previous models have used the chromatin landscape present when the TF is bound in order to infer binding intensity [8] or infer binary binding events [10], [11] (see Discussion).

**Figure 5:**
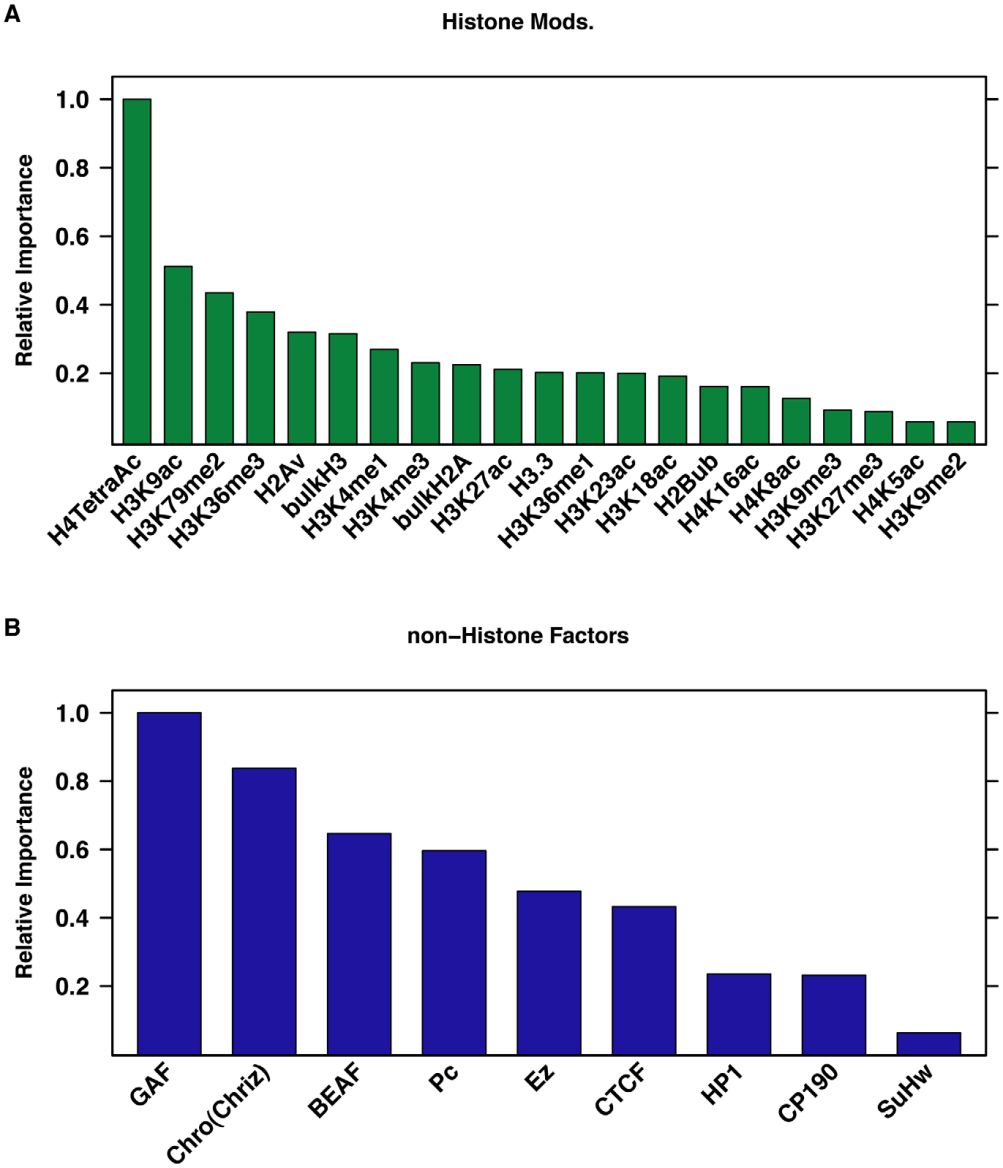
Histone acetylation and GAF occupancy are important covariates in predicting HSF binding intensity. Plotted are the relative values of the sums of the coefficients associated with all rules that reference each covariate in the rules ensemble [20]. Results are shown for (A) the histone variant and modification model and (B) the non-Histone factor model.

Our data and modeling indicated that the presence of active chromatin features, such as histone acetylation and DNase I hypersensitivity, had a significant influence on the predictive power of the model, while repressive features had minimal influence (Figure S9). DNase I hypersensitivity was a strongly predictive covariate in the model when used in a simple linear regression model (Figure 4), or in combination with histone modification and non-histone factor covariates in the rules (Figure S9E–S9G, S9J, S9K, and S9M). Tetra acetylation of H4 and H3K9ac were the most informative histone marks in the model that used histone variants and histone modifications as covariates (Figure 5A). GAGA associated factor (GAF), which has a proposed role in permitting HSF binding [21], was the most influential factor in the HSF binding prediction model that considered all chromatin-binding factors (Figure 5B).

### Defining genome-wide DNA accessibility by chromatin composition

The analysis above indicates that DNA accessibility, as measured by DNase I hypersensitivity, is a primary determinant of binding intensity. Previous studies have similarly shown that TF binding sites correlate strongly with DNase I hypersensitive sites [8], [10], [11], [22]. For instance, histone acetylation causes local chromatin decondensation by reducing the ionic interactions between lysine residues and DNA and promotes accessibility, but the extent to which combinations of histone marks and TFs act together to dictate chromatin accessibility is not known. Therefore, it is of interest to see whether DNA accessibility can be predicted from specific features of the chromatin landscape, such as histone modifications and non-histone chromatin bound factors. In addition, accurate predictions of DNA accessibility would be of practical use, because direct measurements are often not available.

**Figure 6:**
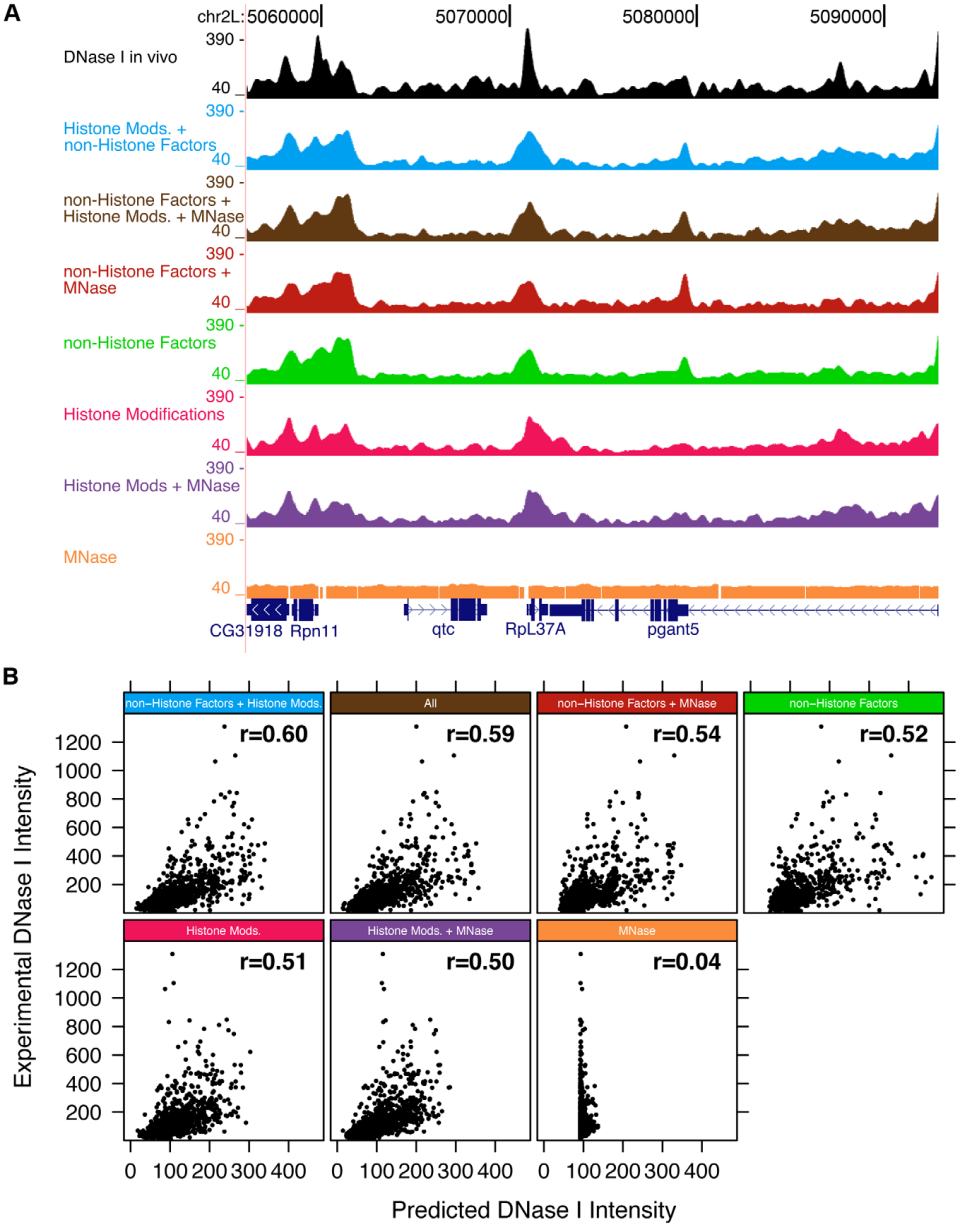
DNase I hypersensitivity can be inferred using histone marks and MNase data. A) The intensity of DNase I hypersensitivity landscape is inferred by models (colors) that use histone modification profiles, non-histone factor profiles, DNase I data and MNase-seq data. B) The experimentally determined DNase I hypersensitivity data is plotted against inferred intensity for the various models. The Pearson correlation for each model is shown.

To address this question, we applied our rules ensemble framework to predict DNase I hypersensitivity (the best available proxy for DNA accessibility) from ChIP-chip data for histone features, non-histone chromatin bound factors, MNase data and combinations of these covariate pools (Figure 6). Tetra-acetylation of H4 and H3K9 acetylation were most influential in the model that uses histone modifications, bulk histone and histone variant intensities (Figure S10E); the correlation coefficient for this model using the test data is 0.51 (Figure S11B). The model that uses non-histone factor ChIP-chip data obtains a correlation of 0.52 (Figure S11B), which is consistent with TFs having characteristic DNase I hypersensitivity footprints [10], [11]. The model that combines both histone data and non-histone data into a rules model performs the best on the test set, with a correlation of 0.60 (Figure S11B). Repressive histone marks appear to contribute little to generating the DNase I hypersensitivity pattern (Figure S10) and the lack of active chromatin marks appears to be sufficient to package DNA into inaccessible units. These models reinforces the notion that the biochemical composition of chromatin permits DNase I hypersensitivity and quantifies the contributions individual modifications, and combinations thereof, make to DNase I hypersensitivity (Figure S11). As more and higher-resolution genome-wide data becomes available, these models will be refined.

### Dissection of the Heat Shock Element

PB–seq provides the opportunity to model the sequence-dependent binding preferences of a purified TF genome-wide and independent of chromatin or other factors. In the case of HSF, the consensus binding site is well characterized and consists of three pentamers, ÒAGAAN NTTCT AGAANÓ, (here denoted pA, pB, and pC), each bound by a monomer of the HSF homotrimer. Note that the consensus sequences for pA and pC are identical, while the one for pB is their reverse complement. Of course, the consensus HSE is a crude summary that ignores subtleties in the base preferences at each position. A position-specific scoring matrix (PSSM) provides a somewhat improved description but still ignores dependencies between positions within the binding site. We sought to use genome-wide binding sites from PB–seq to produce an improved model for the sequence preferences at HSEs.

We began by computing the mutual information for all pairs of HSE positions based on the identified in vitro binding sites. We found negligible evidence of correlated base preferences between positions, but we did observe that some pentamers within PB–seq peaks adhered closely to the consensus motif while others did not. This led us to formulate a probabilistic model that allows each pentamer in an HSE to closely match the consensus (“strict”) or diverge from it more substantially (“relaxed”), and considers all possible combinations of pentamer composition (Figure S12). More specifically, we described each of the three pentamers using a two-component mixture model, with a latent variable indicating “strict” or “relaxed” binding preferences, and estimated the joint distribution of these three latent variables from the data.

The model parameters—the position-specific nucleotide probabilities and prior distribution for the combinations of strict/relaxed pentamers—were estimated from the data by maximum likelihood using an expectation maximization algorithm (see Methods). In fitting the model, we considered only the 1309 isolated HSEs, sequence elements that were at least 200 base pairs away from any other degenerate HSE motif, to avoid complications arising from overlapping HSEs. The model fit the data substantially better than did a simple PSSM (lnL = −15442 vs. lnL = −15673 for the PSSM; Akaike information criterion [AIC] = 15636 vs. AIC = 15763 for the PSSM), suggesting that it effectively captures important dependencies between positions.

**Figure 7:**
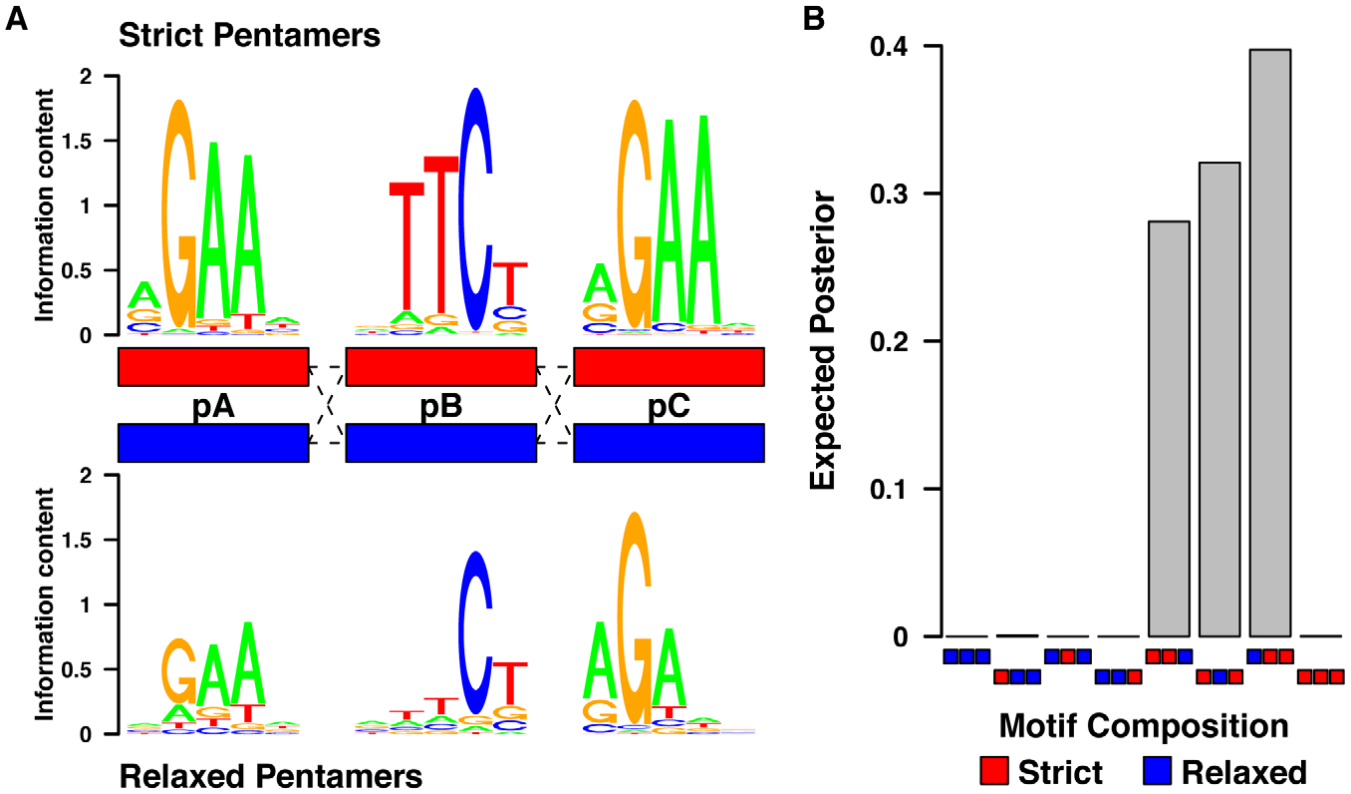
Pentamers within the HSEs are dependent upon their consensus match and also their position relative to the other pentamers. A) The mixture model defines each pentamer within the HSE as strict or relaxed depending upon how well it conforms to the canonical HSE. Note that the position of relaxed pentamers strongly influences their composition. B) A probabilistic sequence model reveals that the presence of two strict (red) and one relaxed (blue) pentamer provides the best explanation of the data.

Based on the estimated model parameters, we computed a posterior probability distribution over all combinations of pentamer stringency and order for each HSE (Methods; Figure 7B). These values were averaged across HSEs to obtain expected genome-wide fractions of HSEs having each of the strict/relaxed pentamer combinations. We found that binding sites with strict pB and pC, and relaxed pA, were most frequent (an expected 38% of sites), indicating that this configuration is preferred (Figure 7B). The next most frequent configurations were a relaxed pB flanked by a strict pA and pC (33%), and a strict pA and pB combined with a weak pC (29%). Interestingly, combinations of three strict pentamers occur at negligible frequency. Indeed, only 5 out of 1309 isolated genomic HSEs matched the consensus sequence exactly, while 148 differed from it by a single mismatch. Configurations with at most one strict pentamer were also rare. Together, these results indicate that the biophysical interactions of the pentamers within the binding sites are critically dependent upon their composition and position relative to the other pentamers in an HSE.

While the three estimated strict pentamer matrices were similar (Figure 7A top), the relaxed matrices showed substantial differences with respect to each other (Figure 7A bottom). For example, the relaxed pA matrix indicates that 70–80% of HSEs containing a weak pA have the consensus base at positions two, three and four. In contrast, position 12 in pC (the analog of position 2 in pA) almost invariably contains a G in all HSEs, while positions 7 and 8 in pB (analogous to positions 3 and 4 in pA) have only modest base preferences in HSEs containing a weak pB. This analysis indicates that each monomeric HSF/pentamer interaction has distinct biophysical properties within the context of the broader HSF/HSE interaction.

We also devised a simplified model, with a single strict matrix shared by all three pentamers, and a single relaxed matrix obtained by applying a “dampening” factor to the strict matrix (Figure S13, Methods). This model further supports the strict/relaxed pentamer split (lnL = −15908 vs. lnL = −16048 for a single-monomer PSSM; and AIC = 15952 vs. AIC = 16078), although both the full model and the full PSSM fit the data better (lower AIC). Moreover, not only was the simplified model still able to reproduce the posterior distributions over pentamer configurations of the full model, but it was also able to replicate synthetic patterns from simulated data (Figure S14).

Finally, the preference for single pentamer degeneracy was also observed independently by comparing the pentamer-specific KL-divergence in PSSMs obtained from subsamples of HSF bound peaks (Figure S15; Methods).

## Discussion

The PB–seq technique combined with EMSA and competition assays provides a straightforward, yet versatile and powerful framework for characterizing all potential binding sites in a genome, regardless of tissue specificity, developmental stage, or environmental conditions. Comparing in vitro and in vivo binding profiles, in the context of pre-induction genomic chromatin landscape, revealed DNase I hypersensitivity, H4 tetra-acetylation, and GAF as critical features that modulate cognate element binding intensity in vivo. Furthermore, DNase I sensitivity was found to be strongly influenced by high GAF occupancy and histone acetylation, while repressive factors were minimally influential in the statistical models. Finally, the full set of potential genomic binding sites provided a rich data set that was used to build more detailed sequence models, which tease apart substructure and features that are lost with traditional PSSM models.

One initially surprising observation from our study was that 40% of the in vivo HSF peaks were not found in vitro. We believe that the limited dynamic range for quantifying in vitro binding affinity may be responsible for the lack of detectable in vitro peaks. Although we quantify in vitro binding over an order of magnitude (40–400 pM), the experimental concentrations of HSF and genomic DNA and our depth of sequencing do not permit the detection of lower affinity HSF binding sites. For instance, only eleven sequence tags would be predicted to underlie a hypothetical 5 nM HSF binding site, and these would not be distinguishable from background. Upon further examination, we find that the composite HSE representing those in vivo binding sites that were not found in vitro is more degenerate than those found using both assays (Figure S16A). Moreover, the in vivo sites that were not found using PB–seq were also more accessible in vivo (Figure S16B), in support of our hypothesis. Performing PB–seq at a range of protein and DNA concentrations, or increasing sequence coverage would expand the dynamic range of quantification by PB–seq.

Other possible explanations for this observation include cooperative interactions with pre-bound chromatin factors, long-range DNA interactions, post-translational modifications of HSF, higher-order chromatin structure, or bridging protein interactions. The influence of DNA modifications and immediate flanking sequence do not contribute to this disparity, since we use large fragments of purified genomic DNA. Bridging protein interactions [17], which do not involve HSF directly binding to DNA, appear not to be responsible for our results because 95% of in vivo peaks encompass at least one HSE near the peak center [6]. However, if other proteins were cooperating with HSF in vivo to enhance HSF binding intensity at low affinity binding sites, then some of these peaks may not be observed in vitro. Since our PB–seq experiment used recombinant HSF in the binding experiments, we would also not capture differences in binding site affinities that are due to post-translational modifications of HSF [16]. To overcome these potential limitations, PB–seq could be adapted to include known bridging/cooperative factors and proteins could be purified from in vivo sources to capture indirect or modification-dependent interactions.

The notion that motif accessibility is driving inducible TF binding in vivo is supported by independent studies of distinct TFs: STAT1, HSF, glucocoticoid receptor (GR), and GATA1 [6], [22]–[24]. These studies show that the chromatin landscape prior to TF binding influences inducible TF binding. In the first study, it was found that a large fraction of STAT1 induced binding sites contained H3K4me1/me3 marks prior to interferon-gamma (IFN-γ) induced STAT1 binding [23]. Our group previously found that inducible HSF binding sites are marked by active chromatin compared to sites that remain HSF–free [6]. A more recent study has shown that inducibly bound GR sites are marked by DNase I hypersensitive chromatin prior to GR binding [22]. Likewise, the permissive chromatin state at GATA1 binding sites is established even in GATA1 knock out cells [24]. While these correlations are instructive, no previous attempt has been made to model inducible TF binding using biological measurements of chromatin landscape present prior to TF binding. Recent models have successfully inferred TF binding profiles using DNA sequence and chromatin landscape data, generated at the same time the TF is bound [8]–[11]. However, these models do not distinguish between the influence TFs have upon local chromatin and the chromatin features that permit TF binding. In contrast, we modeled the changes between HSF in vitro binding (PB–seq) and in vivo binding (ChIP-seq) landscapes as a function of the non-heat shock chromatin state. This produced a quantitative model describing the important features that modulate the in vivo HSF binding intensity. Moreover, the use of our rules ensemble model enabled the capture of potential interactions between these chromatin features.

Our study reveals that DNase I hypersensitivity and acetylation of H4 and H3K9 are strong predictors of inducible HSF binding intensities, however the molecular events and factors that precede TF occupancy to maintain accessible chromatin remain poorly characterized. For instance, the degree to which pioneering factors or flanking DNA sequence, individually or in combination, maintain or restrict accessibility remains unclear. A recent study highlights the biological consequences of maintaining the inaccessibility of TF binding sites, in order to repress expression of tissue-specific transcription factors in the wrong tissues. The authors found that ectopic expression of CHE-1, a zinc-finger TF that directs ASE neuron differentiation, in non-native *C. elegans* tissue is not sufficient to induce neuron formation [25]. However, combining ectopic CHE-1 expression with knockdown of *lin-53* did modify the expression patterns of CHE-1 target genes in non-native tissue, effectively converting germ line cells to neuronal cells [25]. LIN-53 has been implicated in recruitment of deacetylases, and deacetylase inhibitor treatment mimics *lin-53* depletion, suggesting that LIN-53 is actively maintaining CHE-1 target sites inaccessible in germ cells.

Alternatively, functional TF binding sites could be actively maintained in the accessible state. HSF binding within ecdysone genes has a functional role in shutting down their transcription [14], and activating ecdysone-inducible genes containing inaccessible HSEs causes chromatin changes that are sufficient to allow HSF binding [6]. In this special case of HSF–bound ecdysone genes, active transcription and the corresponding histone marks are mediating access to HSEs, in order for HSF to bind and repress transcription upon heat shock. A more recent study has shown that activator protein 1 (AP1) actively maintains chromatin in the accessible state, so that GR can bind to cognate elements [26].

Although TF accessibility to critical genomic sites appears to be actively maintained, many binding sites may be a non-functional result of fortuitous TFBS recognition. It has long been hypothesized that the binding affinities for TF/DNA interactions are sufficiently strong to allow promiscuous binding at the cellular concentrations of TFs and DNA [27], [28]. There are roughly 32,000 HSF molecules per tetraploid S2 cell [29] and the dissociation constants for trimeric-HSF/HSE interactions are in the picomolar range (Figure 2E); therefore much of the in vivo HSF binding may be non-functional promiscuous binding. Additional investigation will further illuminate the role of chromatin context in TF binding and the mechanisms by which programmed developmental or environmental chromatin changes permit or deny TF binding.

Elucidating the rules that govern accessibility is essential for predicting in vivo occupancy of TFs. Diverse transcription factors [7], from a broad spectrum of organisms [22], bind their sequences based on site accessibility. We found that chromatin accessibility as measured by DNase I hypersensitivity could be inferred using ChIP-chip data for various histone modifications and transcription factors. Although our model can infer accessibility based on chromatin composition, the mechanism by which accessibility originates is not addressed. Previous studies have shown that activators, such as HSF, glucocorticoid receptor, and androgen receptor bind to their cognate sites and direct a concomitant increase in local acetylation, DNase I hypersensitivity, and nucleosome depletion [6], [22], [30], [31]. Androgen receptor also acts to position flanking nucleosomes marked by H3K4me2 [31]. These post-TF binding chromatin changes that occur are the result of acetyltransferase and nucleosome remodeler recruitment, both of which functionally interact with activators. For instance, both GR and GATA1 interact with the nucleosome remodeling complex Swi/Snf [32], [33]. Concomitant increases in locus accessibility likely allow large molecular complexes such as RNA Pol II and coactivators to access the region that in turn can reinforce and maintain active and accessible chromatin.

Thorough biophysical characterization of TF binding site properties is critical for accurate predictions of TF binding sites, underscoring the need for more complete models of TF binding. While the commonly used PSSM model makes the assumption of base independence, recent work has revealed that richer models providing for interactions between positions are necessary [34], [35]. Our model captures critical features of the HSF/HSE interaction that are lost with simpler computational models, namely the interdependencies between the sub-binding sites of each HSF monomer. Consistent with our model, a series of in vitro experiments with *S. cerevisiae*, *D. melanogaster*, *A. thaliana*, *H. sapien* and *D. rerio* HSFs indicate that HSF from each of these species can bind to discontinuous HSEs containing canonical pentamers that contain intervening five base pair gaps [36], [37]; interestingly, however, *C. elegans* HSF strictly binds to continuous HSEs that do not contain gaps [36]. The complex interactions between positions within a binding site are a critical aspect of inferring whether a polymorphism or mutation affects TF binding. These features should prove useful in providing degenerate HSE sequences for optimal co-crystallization of trimeric HSF and DNA and inferring changes in DNA sequence that affect HSF binding within and between species.

In conclusion, the data and models presented here reinforce both the importance of chromatin landscape in modulating in vivo TF binding intensity and how genome wide, chromatin free, binding assays contribute to the understanding of TF sequence binding specificity.

## Methods

### Cloning and purification of recombinant HSF

*Drosophila* HSF was N-terminally tagged with glutathione s-transferase and a tobacco etch virus (TEV) protease cleavage site. The C-terminus of the recombinant HSF was fused to the 3xFLAG epitope. Recombinant HSF was purified from *E. coli* with glutathione resin as previously described [38], with the following modifications: HSF–3xFLAG elution was achieved by addition of 6xHistidine tagged TEV protease and TEV protease was cleared from the HSF preparation using a Nickel-NTA column. Densitometry was used to show that the HSF protein preparation was 40% full length HSF–3xFLAG, and known amounts of bovine serum albumin (BSA) were used to quantify the HSF (Figure S1).

### Band shift assay

Serial two-fold dilutions of recombinant HSF, from 3 nM (1.5 nM for the 221 pM HSE) to 23.3 pM, was incubated with 200 attomoles of radiolabeled dsDNA containing modestly degenerate HSEs (chrX:3380775–3380824 (224 pM), chr2L:5009892–500994 (42.7 pM), chr2R:3529792–3529841 (308 pM), chr3L 13470978–13471009 (221 pM), and chr3L:4073542–4073591 (97.5 pM)) and allowed to come to equilibrium for 30 minutes in a total of 10 µl of 1xHSF binding buffer (20 mM HEPES pH 7.9, 10% glycerol, 1 mM EDTA, 4 mM DTT, 3 mM MgCl2, 100 mM NaCl, 0.1% NP-40, and 300 µg/ml BSA) at room temperature. Binding reactions were loaded in a 3% agarose TBE (10 mM Tris-HCl pH 8.0, 25 mM boric acid, and 1 mM EDTA) gel and electrophoresed at 50 Volts for 2 hours. The HSF–bound probe and free probe were quantified by densitometry and the dissociation constant, Kd = ([A][B])/[AB], was estimated using a non-linear least squares method on the function [AB]/[A]_total_ = [B]/([B]+Kd) where [AB]/[A]_total_ is the measured shifted fraction and [B] is the free HSF trimer concentration.

### PB–seq: Genomic in vitro binding experiment

We incubated 600 pM HSF and 2500 ng genomic DNA (sonicated to 100–600 bp fragment size as previously described [6]) in 1500 µl final volume of 1xHSF binding buffer and let it come to equilibrium for an hour at room temperature. We added 20 µl ANTI-FLAG M2 affinity gel for 10 minutes and washed 8 times with 1xHSF binding buffer to remove unbound DNA, 3xFLAG peptide was added to a final concentration of 200 ng/µl to specifically elute HSF and HSF–bound DNA. The mock IP was done in the absence of recombinant HSF. We attribute the in vitro binding assay's low background to the design of the experiment. Since recombinant C-terminally 3xFLAG tagged HSF was used, the HSF–associated DNA could be specifically eluted by the addition of excess 3xFLAG peptide. In contrast, standard ChIP protocols rely on non-specific elution of all protein and DNA that binds the resin.

### Illumina library preparation

The sample preparation was as previously described [6], except that 15 rounds of amplification were performed in this case.

### PB–seq HSF peaks and HSE sites

The PB–seq reads were aligned to the *Drosophila* Genome (BDGP R5/dm3) using BWA (v 0.5.8c) [39]. We obtained 5,052,425 uniquely aligned reads for replicate one, 4,694,846 for replicate two and 5,410,049 for the mock. Files that contain raw sequence data and uniquely aligned reads were deposited into NCBI's Gene Expression Omnibus (GEO) [40], accession number GSE32570.

We called peaks using MACS (v 1.3.7.1) [41], both for each individual replicate and for the merged set, using a tag size of 55 bp, a starting bandwidth of 100 bp and an appropriate genome size. After experimenting with several p-value thresholds, we selected a value of *p* = 0.01, which achieved a good tradeoff between maximizing the number of called peaks and ensuring consistency between replicates. Our results were largely unaffected by the ‘mfold’ parameter (the threshold for fold enrichment relative to background for inclusion in the peak model), so we left this parameter at its default value.

To improve our sensitivity in binding site detection, we made use of an ensemble of position weight matrices (PSSMs), rather than a single matrix. We sampled 10,000 sets of 100 peaks and used the program MEME [42] for motif discovery in each set. As input, MEME was given the 100 bp sequence centered at each peak summit. We used a fixed motif width of 14 bp, a second order background Markov model estimated from the entire peak set, and the ‘zoops’ model (zero or one site per sequence) with the restriction that at least 75% of the sequences must contain a site. The resulting PSSMs were compared by KL-divergence against the canonical monomer PSSM (four base pair unit with consensus AGAA) estimated from the previously published in vivo high-confidence HSF binding sites detected by ChIP-seq [6]. In each PSSM, one of the three monomers had on average about twice the KL-divergence as the other two. Figure S15 shows a scatter plot of the KL-divergence of the PSSMs in the ensemble

Each peak was scanned for matches to all PSSMs in the ensemble, allowing for overlapping sites. The score at each position was taken to be the maximum score across the ensemble. Peaks were split into three groups by GC% quantile, and for each group a 10 kbp sequence was simulated from a second order Markov model, which was then used to estimate the FDR associated with the score.

In our context, an appropriate FDR threshold should strike a balance between recapitulation of in vivo results and limiting the number of spurious binding sites. In vivo results are defined by high-confidence peaks, which are ChIP-seq peaks that were called by two peak calling programs and have a corresponding binding site sequence underlying the peak [6]. Whereas, spurious sites are accounted for by limiting the average number of HSE clusters per peak (set of potentially overlapping HSE no more than 10 bp apart from each other). Due to the repetitive nature of the HSE, a cluster is a better representative than a single site of a functional binding locus. We chose a 20% FDR threshold, which maximizes the fraction of peaks having a single HSE cluster while ensuring that a large fraction (97%) of the high-confidence in vivo peaks contain HSEs. This threshold resulted in 3735 clusters (71% with a single HSE, 20% with two HSEs overlapping by 10 bp, ∼5% with two HSEs overlapping by 5 bp; see Figure S17).

The final set of HSE clusters was obtained by combining data from the two experimental replicates. First, a set of genomic regions was identified by intersecting the peaks from the two experimental replicates, and retaining only those peaks for which the two replicates were in close agreement (>80% of reads fall in the overlapping region). We then identified the 2896 HSE clusters that fell in these regions (∼77% of all clusters).

### HSE cluster intensity

The problem of measuring the intensity of each peak is complicated by the fact that some peaks contain multiple, closely spaced clusters, whose contributions are difficult to disentangle. Furthermore, peaks often include trailing edges that are dominated by the background signal. To address these concerns we experimented with various measures of intensity based on the output produced by MACS (wig files giving shifted read counts in 10 bp windows) as well as the reported ‘bandwidth’ *B*. We considered three measures, applied to a window of radius *B* centered at each cluster: maximum read count, read count sum, and an “integrated” read count based on a biweight kernel (which produces a curve at each peak that is similar to the one implied by the peak model used by MACS). We selected the biweight kernel measure, which does the best job of handling closely spaced clusters (see Figure S18).

### Computing K_d_ values for all genomic HSE sites

We assume that each HSE site *i* is at approximately the same initial concentration in the experiment ([*HSE_i_*]*^initial^* = *C*). Furthermore, all sites compete to bind a shared amount of free HSF, with the remaining unbound concentration denoted by [*HSF*]. At the end of the experiment, a fraction of site *i* is bound, with concentration [*HSE_i_* : *HSF*], and the remainder is unbound, with concentration [*HSE_i_*]. The dissociation constant for a particular HSE site is therefore given by:
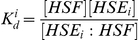



The bound HSE concentration is measured by the PB–seq experiment in terms of the number of reads at element *i* (*R_i_*). This leaves two unknown quantities, [*HSF*] and [*HSE_i_*], in units of read counts. The first of these unknowns, [*HSF*], can be eliminated by considering instead the relative *K_d_* with respect to a known reference value (for an HSE present in the experiment).

To solve for [*HSE_i_*], we express this quantity as the difference between the initial concentration *C* and the measured bound concentration:
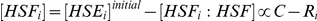
By substituting the expression for *K_d_^i^* (above) and dividing by the *K_d_* value for the reference HSE, *K_d_^ref^*, we obtain an expression with a single unknown, *C*:
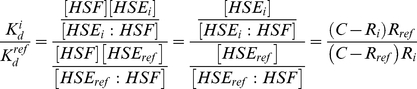
With the use of a reference dissociation value for a second HSE, we can solve for *C* and obtain estimates of the dissociation constants for all other HSE sites for which read counts are available. Replacing *K_d_^i^* and *R_i_* by the corresponding values for the second reference HSE and solving for *C*:
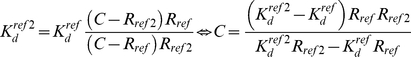



### Heat Shock Element model

Our probabilistic model for HSEs was designed to capture interactions among the binding preferences of the three monomers that form the HSF homotrimer. The model consists of three PSSM-based submodels corresponding to the three 5 bp sequences (pentamers) that are bound by the HSF monomers. Each of these submodels is defined by two PSSMs, one ‘strict’ and one ‘relaxed’. These three submodels allow for eight possible combinations of strict and relaxed pentamer binding. Within each pentamer the positions are considered independent, as in standard PSSM models.

Formally, let a candidate 15 bp HSE sequence *X^k^* be composed of random variables *X_i,j_^k^* where *i* is the pentamer index and *j* is the base position within that pentamer. Additionally, let each sequence have an associated unobserved random variable *Y^k^* which indicates which of the eight combinations of strict/relaxed distributions are applied the corresponding *X_i,j_^k^* (Figure S12). For simplicity, our model definition assumes that the middle monomer sequence has been reverse complemented and is therefore in the same orientation as the outer monomer binding sequences. We considered two versions of the model: a sparsely parameterized ‘constrained’ version and a more parameter-rich ‘expanded’ version, as described below.

#### Constrained version

This version of the model assumes that the three monomers share the same strict and the same relaxed PSSM-based sequence distributions. In addition, it assumes that the relaxed PSSM is defined as a more degenerate version of the strict PSSM. This is accomplished by means of a single ‘degeneracy’ parameter, which ‘pulls’ the nucleotide distribution at each position toward the uniform distribution. Specifically, the nucleotide distribution at position *j* of pentamer *i* is defined as:
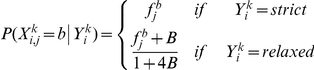
where *f_j_^b^* is the probability of observing nucleotide *b* at position *j* of the monomer and *B* is the free parameter controlling how close to an uniform distribution the relaxed version is.

To estimate the model parameters from the HSE sequence data, we first held *B* fixed and then estimated the nucleotide frequencies and the prior probabilities of each strict/relaxed monomer combinations through Expectation Maximization (EM). A grid search was then used to find the value of *B* that maximized the model likelihood.

Estimating the model parameter updates for EM is simple for the prior but slightly more complicated for the nucleotide frequencies due to the interdependency between the strict and relaxed distributions. Nevertheless, it can be solved by using a Lagrange multiplier together with the derivatives of the expected complete log-likelihood. This produces an estimator that depends on the Lagrange multiplier and requires the use of a root finding method as part of the maximization step of EM. Figure S13C shows the results of the parameter estimation. To initialize the optimization procedure, the nucleotide frequencies were estimated from the high-confidence in vivo HSEs from [6].

#### Simulation study

To test the performance of this model, we estimated unconstrained strict/relaxed matrices from real data and simulated data under various distributions of Y^k^. All of the parameters of the simplified model were then estimated from this simulated data, and the posterior distribution of Y^k^ under the model was compared with the values used for simulation (Figure S14).

#### Expanded version

This version of the model allows for completely separate PSSMs for the three pentamers, and completely separate strict and relaxed versions of each of these models. It has 5×3×3×2 = 90 PSSM parameters plus seven free parameters for Y^k^, for a total of 97 free parameters. Parameter estimation is again accomplished by expectation maximization, but in this case the parameter updates are trivial.

### Chromatin effect and DNase I hypersensitivity models

The chromatin effect and DNase models are rule ensemble models, estimated using the RuleFit R package. This package was also used to estimate the relative importance of the model covariates. The covariates were obtained from modENCODE tracks, taking the mean value over a 200 bp window centered on the target point. Furthermore, these data were filtered to contain only points that had a value for every covariate used.

#### Chromatin effect model

This model estimates the ratio between the in vivo and in vitro intensities at each site from a set of chromatin covariates. Ratio values were obtained from the measured intensities using the in vitro HSE cluster coordinates. The set of HSE clusters was pre-filtered by finding a threshold on the in vitro intensities that approximately minimized the differences between experimental replicates. The threshold was obtained as follows: 1) for each candidate in vitro threshold value, collect the mean absolute difference between experimental replicates for the ratios computed using in vitro intensities above that value; 2) compute the mean of the values collected in the previous step; 3) pick the first in vitro threshold that falls below the mean.

The model was estimated on the selected HSE clusters using different combinations of chromatin covariates. For each particular combination, an estimated Pearson correlation value was obtained from ten-fold cross validation. Furthermore, to obtain the figures presented in this paper, the data was split into 60% training data and 40% test data. The model obtained on the training data was used to make the test data predictions shown in the figures and the corresponding Pearson correlation.

#### DNase I hypersensitivity model

The data set used for this model was independent of the HSE clusters. 10K points were randomly sampled from across the genome with the restriction that the points did not fall within the regions shown in the figures presented in this paper or within 200 bp of the HSE cluster sites. These 10K points were used as a training set to build the model used to make the predictions for the browser tracks and at the HSE cluster regions. They were also used to estimate the Pearson correlation via ten-fold cross validation.

#### Prediction tracks

To produce the in vivo intensity prediction tracks, the chromatin model was applied to a version of the in vitro intensities that were scaled so that they would be comparable to the in vitro intensities. To obtain the scaling factor, we selected the top seven most accessible isolated HSE clusters (as measured by DNase hypersensitivity) that had a significant read count. The reason for these restrictions is that highly accessible sites should be good proxies for sites that are not being influenced by chromatin effects and the sites with significant in vitro intensity should produce better estimates of the in vivo to in vitro ratio used for scaling (see Figure S4 for point choices).

The browser tracks were produced by collecting values in 50 bp steps with a 100 bp window average and applying the respective model and scaling (if needed). Values were then smoothed with a Gaussian kernel having a 100 bp bandwidth.

## Supporting Information

Figure S1 HSF purification and quantification. A) Purified full-length HSF (arrow) was estimated to be 40% pure as quantified by a silver stained gel and densitometry. B) A silver stained gel using known concentrations of BSA (10 ng/µl, 5 ng/µl, 2.5 ng/µl, 1.25 ng/µl) was used to quantify the stock concentration of purified full-length HSF (arrow) at 1.9 ng/µl. Note that one gel is shown, but intervening lanes were removed for simplicity.
(TIF)

Figure S2 A) The mobility of the constant 200 attomole HSE probe shifts into a trimeric-HSF:HSE complex as increasing HSF is added. There is no HSF in the left-most lane, the right-most lane contains 3 nM HSF (1 nM trimeric HSF), and the intervening lanes contain two-fold serial dilutions of HSF. B) A hyperbolic curve based on the Kd equation (see Methods) was modeled using the band shift data, indicating a Kd of 97.5 pM (95% confidence interval of 59.8–158 pM). C) The constant 200 attomole HSE probe shifts into a trimeric-HSF:HSE complex as increasing HSF is added. There is no HSF in the left-most lane, the right-most lane contains 1.5 nM HSF (500 pM trimeric HSF), and the intervening lanes contain two-fold serial dilutions of HSF. D) A hyperbolic curve based on the Kd equation (see Methods) was modeled using the band shift data, indicating a Kd of 221 pM (95% confidence interval of 197–250 pM). E) This panel has the same description as panel A. F) A hyperbolic curve based on the Kd equation (see Methods) was modeled using the band shift data, indicating a Kd of 308 pM (95% confidence interval of 214–448 pM).
(TIF)

Figure S3 Each panel shows smoothed 95% confidence intervals (CI) (dotted lines) for the estimated genomic Kd values (blue lines). The red and green points correspond to the Kd values determined by the EMSA assays. Error bars indicate 95% confidence intervals (CIs), as estimated in the non-linear regression (see Methods). Red points indicate those used as references to compute the genomic Kd values in each panel. The CIs shown in panels A1, B1, A2 and B2 were estimated by propagating various sources of uncertainty through our formula for estimating Kd values, using the first order Taylor expansion approximation. In panels A1 and A2, only the variance associated with the reference Kd points was considered, whereas in B1 and B2 the variance associated with the site intensity estimates was also used. At each binding site in the genome, the variance in intensity was estimated analytically from the two PB–seq replicates, after quantile normalization of the PB–seq replicate intensities to remove systematic biases. In panels C1 and C2, the CIs were computed by sampling the reference Kd values from normal distributions corresponding to their respective CIs and by selecting site intensities at random from one of the two PB–seq replicate values (again after quantile normalization). To account for the uncertainty associated with the choice of reference points, we show the CIs based on the two best EMSA points in the top panels and those based on the two worst EMSA points in the bottom panels.
(TIF)

Figure S4 These data points (HSE cluster sites) were used to determine the scaling factor between in vivo and in vitro binding intensities in Figure 1 and Figure 3. The top left plot shows how the in vivo to in vitro intensity ratio varies with the number of points included; dashed line signals the final choice of seven points. Scatter plots show the top 30 data points (HSE cluster sites) with the highest DNase I signal, against their in vivo and in vitro intensity values; black indicates the seven chosen points. The points with higher DNase I hypersensitivity offer the best choice for unbiased scaling.
(TIF)

Figure S5 This UCSC genome browser shot provides additional examples of in vivo prediction of HSF binding intensity using chromatin and PB–seq data.
(TIF)

Figure S6 The experimentally determined ratio between in vivo ChIP-seq HSF intensity and in vitro PB–seq intensity is plotted against the predicted in vivo/actual PB–seq ratio. The Pearson correlation for each model is shown.
(TIF)

Figure S7 The bar graphs indicate the Pearson correlation of predictions versus experimental measures for each model used to predict the in vivo/in vitro binding intensity ratio (Rul: Rules Ensemble model, Lin: linear regression model). The correlations for both the training data (panel A) and the test data (panel B) are indicated.
(TIF)

Figure S8 ROC plots for in vivo HSF binding predictions. In vitro HSE sites were partitioned into bound and unbound cases by applying a threshold to the estimated in vivo intensity values. Three thresholds were considered: a permissive threshold (shown in red; 36% bound), a moderate threshold (green; 24% bound) and a strict threshold (blue; 12% bound). Each panel in the figure represents a distinct covariate set (see panel titles). For each covariate set, the corresponding rules ensemble model was applied to predict the in vivo intensity of the HSE sites. Each site was then classified as predicted to be bound or unbound by applying a threshold to these predicted intensities. These thresholds were varied to produce the Receiver Operating Characteristic (ROC) curves shown. As a baseline, we show predictions based on the scaled in vitro intensities in gray. For each ROC curve, we compute the Area Under the Curve (AUC) as a general measure of prediction performance (higher is better). Notice that the ROC curves are not highly sensitive to the threshold that is applied to the in vivo intensities, but in most cases the ensemble model produces a substantial improvement over the baseline prediction. At the same time, some covariates produce substantially better predictions than others.
(TIF)

Figure S9 For each of the models shown in Figure S6 we show the relative importance [20] of each covariate in the Rule Ensemble model built with each indicated subset of covariates to predict the in vivo/in vitro binding intensity ratio.
(TIF)

Figure S10 The bar graphs illustrate the relative importance [20] of each covariate in the Rule Ensemble model built with each indicated subset of covariates to predict DNase I hypersensitivity.
(TIF)

Figure S11 These bar graphs indicate the Pearson correlation of predicted versus experimentally measured DNase I sensitivity for each DNase I prediction model (Rul: Rules Ensemble model, Lin: linear regression model). The correlations for the training data (panel A) and test data (panel B) are indicated.
(TIF)

Figure S12 The structure of the HSE probabilistic sequence model recapitulates the structure of the HSE. Each hidden variable Y_1_,Y_2_,Y_3_, determines if the respective underlying pentamer bases are drawn from a strict base distribution or a relaxed version.
(TIF)

Figure S13 Pentamers within the HSEs are dependent upon their stringency and position relative to the other pentamers. A) A composite pentamer matrix was derived from all pentamers found within PB–seq peaks. B) The strict motif from panel A and a dampening factor from panel C were used to generate a relaxed motif. C) The dampening factor was optimized to generate a relaxed motif that best explained the data. D) A probabilistic sequence model reveals that the presence of two strict and one relaxed pentamer provides the best explanation of the data.
(TIF)

Figure S14 The reduced HSE sequence model predictions are compared for patterns of strict/relaxed pentamer combinations. Three different simulated patterns are shown and are recapitulated by the model.
(TIF)

Figure S15 Scatter plots show similarity of each HSE pentamer to the canonical monomer PSSM. Each point represents a PSSM estimated via MEME by sub-sampling the in vitro peaks identified by MACS. Pattern of the scatter plot shows evidence for pentamer divergence occurring on one pentamer at a time (points are spread following the axis, mainly corresponding relaxed versions of the first and second pentamers).
(TIF)

Figure S16 In vivo HSF binding sites that were either detected or not detected in vitro have distinct properties. A) The composite PSSM for the 40% of HSF binding sites that are only found in vivo exhibits more degeneracy than the PSSM from the sites that are found both in vivo and in vitro. B) The binding sites exclusively found in vivo are generally more accessible, as measured by DNase I signal, than those sites found both in vivo and in vitro.
(TIF)

Figure S17 A) Balance between in vivo recall and number of per peak in vitro HSE is reached at 20% estimated FDR, corresponding to the inflection point for the number of clusters, as well as near maximal recall of high-confidence in vivo sites. B) An HSE (or HSE cluster) is considered isolated if the nearest neighbor is more than 200 bp away. An HSE (or HSE cluster) is considered overlapping if it overlaps with a single other HSE (or HSE cluster); overlaps between more than two HSE (or HSE clusters) are denoted as complex overlaps.
(TIF)

Figure S18 Three different measures were compared for computing HSE cluster intensities: max, sum and bi-weight kernel. A) Each measure was incorporated into a scatter plot of scaled intensities. Max was rejected because it produced a more compressed range of intensity values. B) In comparing the difference in intensities across replicates, the bi-weight kernel approach fares slightly better than the sum. C) The difference in magnitudes given a cluster distance on isolated clusters was compared between measures. For each distance, the isolated clusters are made to overlap an identical copy of themselves and the magnitude difference is computed by comparing the value of the isolated cluster with the partially overlapping, using either the sum or kernel measures. Average values per intensity quartile show that bi-weight kernel measure introduces less error as a function of distance than the sum measure. D) Replicate intensities strongly correlate, as predicted, using the kernel measure.
(TIF)

Table S1 ModENCODE identification number or GEO accession number for each data set used in the paper.
(XLS)
