# Supplementary figures and images for: Accurate Prediction of Inducible Transcription Factor Binding Intensities In Vivo
Source: PLoS Genet. 2012 Mar 29;8(3):e1002610. doi: 10.1371/journal.pgen.1002610 (PMC3315474; doi:10.1371/journal.pgen.1002610)

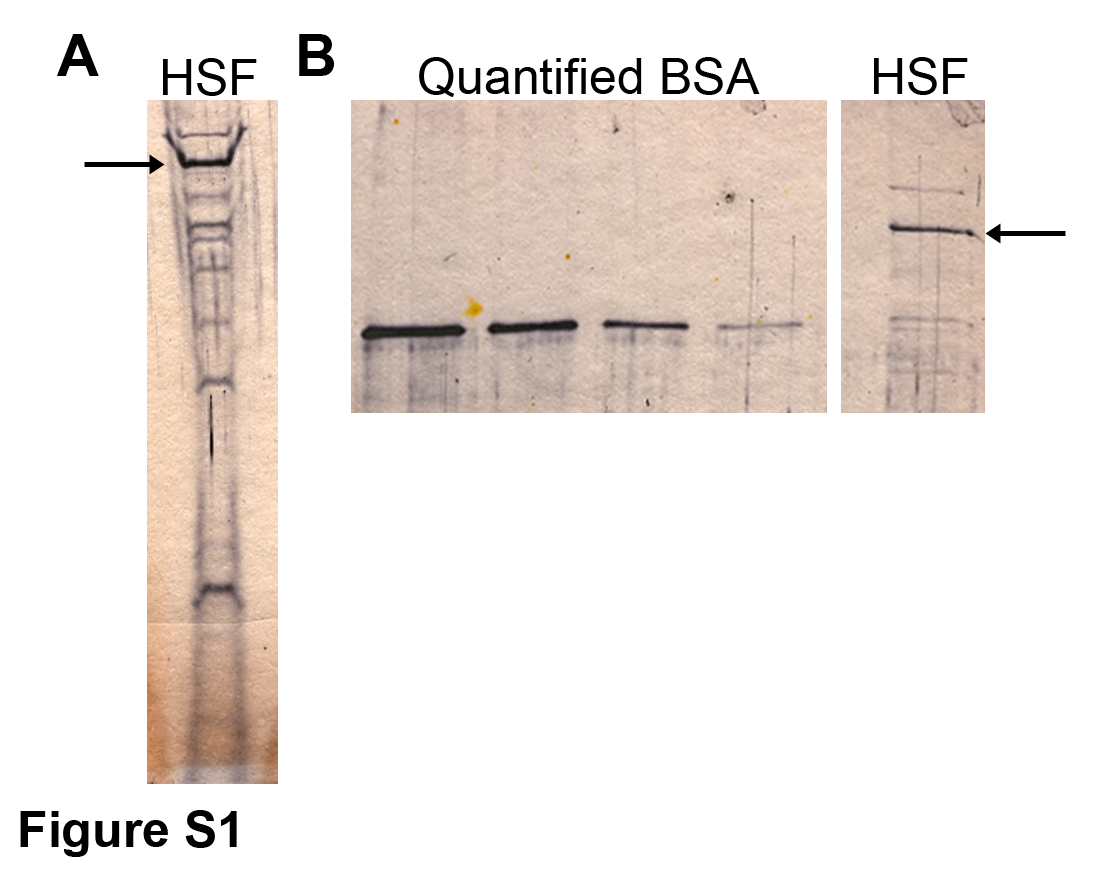

Supplement: Figure S1 — HSF purification and quantification. A) Purified full-length HSF (arrow) was estimated to be 40% pure as quantified by a silver stained gel and densitometry. B) A silver stained gel using known concentrations of BSA (10 ng/µl, 5 ng/µl, 2.5 ng/µl, 1.25 ng/µl) was used to quantify the stock concentration of purified full-length HSF (arrow) at 1.9 ng/µl. Note that one gel is shown, but intervening lanes were removed for simplicity. (TIF) [file pgen.1002610.s001.tif]

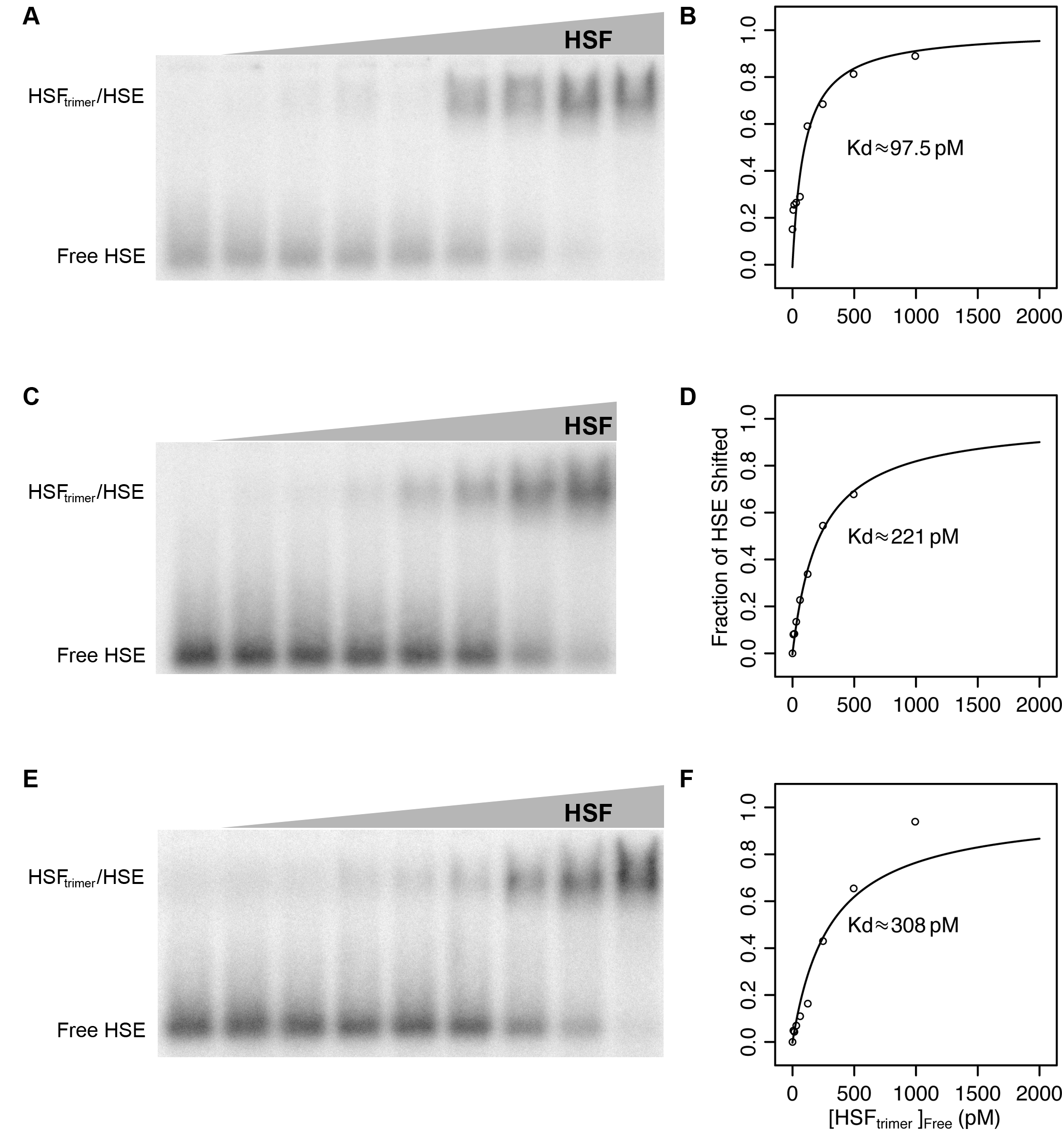

Supplement: Figure S2 — A) The mobility of the constant 200 attomole HSE probe shifts into a trimeric-HSF:HSE complex as increasing HSF is added. There is no HSF in the left-most lane, the right-most lane contains 3 nM HSF (1 nM trimeric HSF), and the intervening lanes contain two-fold serial dilutions of HSF. B) A hyperbolic curve based on the Kd equation (see Methods) was modeled using the band shift data, indicating a Kd of 97.5 pM (95% confidence interval of 59.8–158 pM). C) The constant 200 attomole HSE probe shifts into a trimeric-HSF:HSE complex as increasing HSF is added. There is no HSF in the left-most lane, the right-most lane contains 1.5 nM HSF (500 pM trimeric HSF), and the intervening lanes contain two-fold serial dilutions of HSF. D) A hyperbolic curve based on the Kd equation (see Methods) was modeled using the band shift data, indicating a Kd of 221 pM (95% confidence interval of 197–250 pM). E) This panel has the same description as panel A. F) A hyperbolic curve based on the Kd equation (see Methods) was modeled using the band shift data, indicating a Kd of 308 pM (95% confidence interval of 214–448 pM). (TIF) [file pgen.1002610.s002.tif]

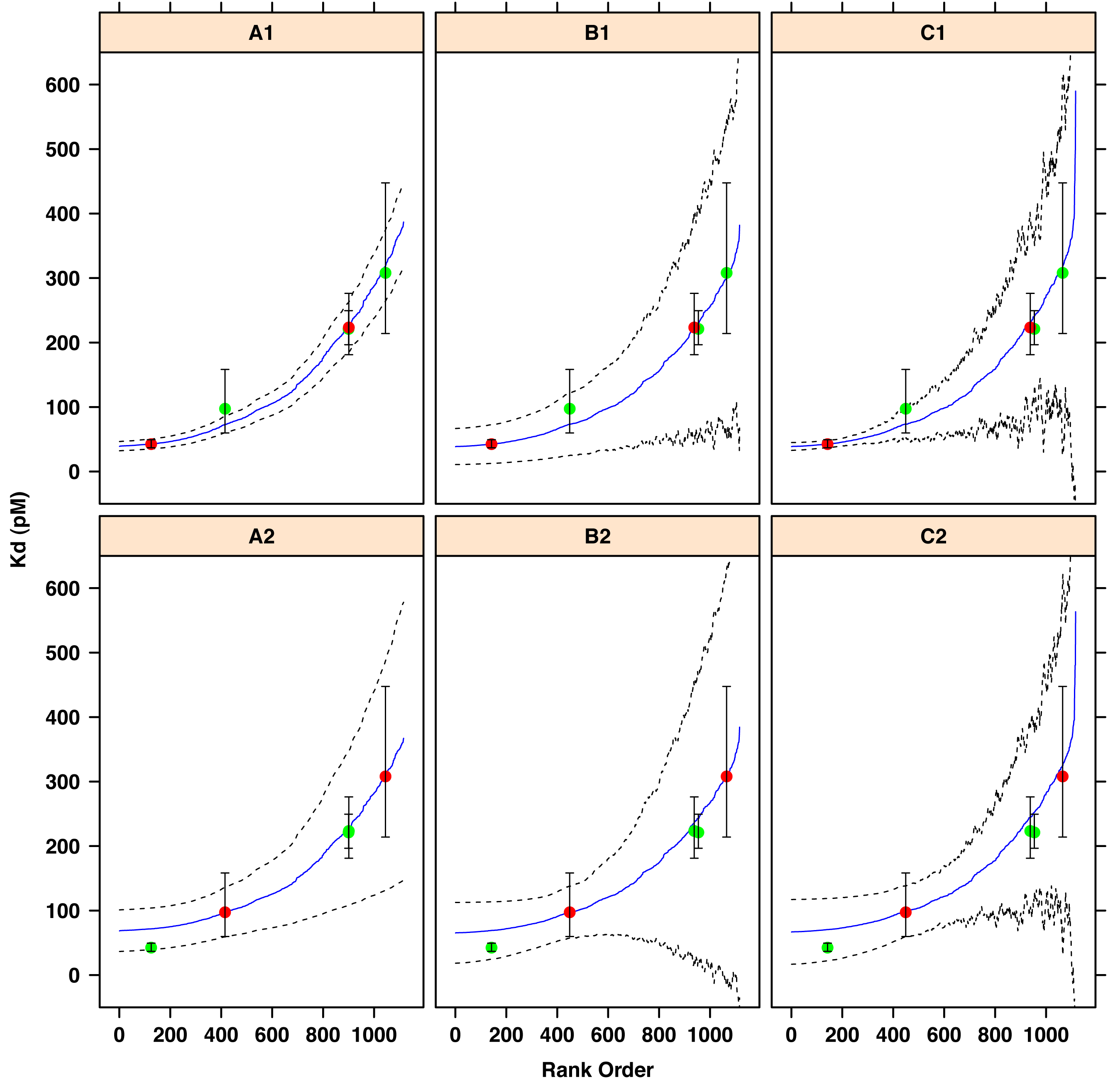

Supplement: Figure S3 — Each panel shows smoothed 95% confidence intervals (CI) (dotted lines) for the estimated genomic Kd values (blue lines). The red and green points correspond to the Kd values determined by the EMSA assays. Error bars indicate 95% confidence intervals (CIs), as estimated in the non-linear regression (see Methods). Red points indicate those used as references to compute the genomic Kd values in each panel. The CIs shown in panels A1, B1, A2 and B2 were estimated by propagating various sources of uncertainty through our formula for estimating Kd values, using the first order Taylor expansion approximation. In panels A1 and A2, only the variance associated with the reference Kd points was considered, whereas in B1 and B2 the variance associated with the site intensity estimates was also used. At each binding site in the genome, the variance in intensity was estimated analytically from the two PB–seq replicates, after quantile normalization of the PB–seq replicate intensities to remove systematic biases. In panels C1 and C2, the CIs were computed by sampling the reference Kd values from normal distributions corresponding to their respective CIs and by selecting site intensities at random from one of the two PB–seq replicate values (again after quantile normalization). To account for the uncertainty associated with the choice of reference points, we show the CIs based on the two best EMSA points in the top panels and those based on the two worst EMSA points in the bottom panels. (TIF) [file pgen.1002610.s003.tif]

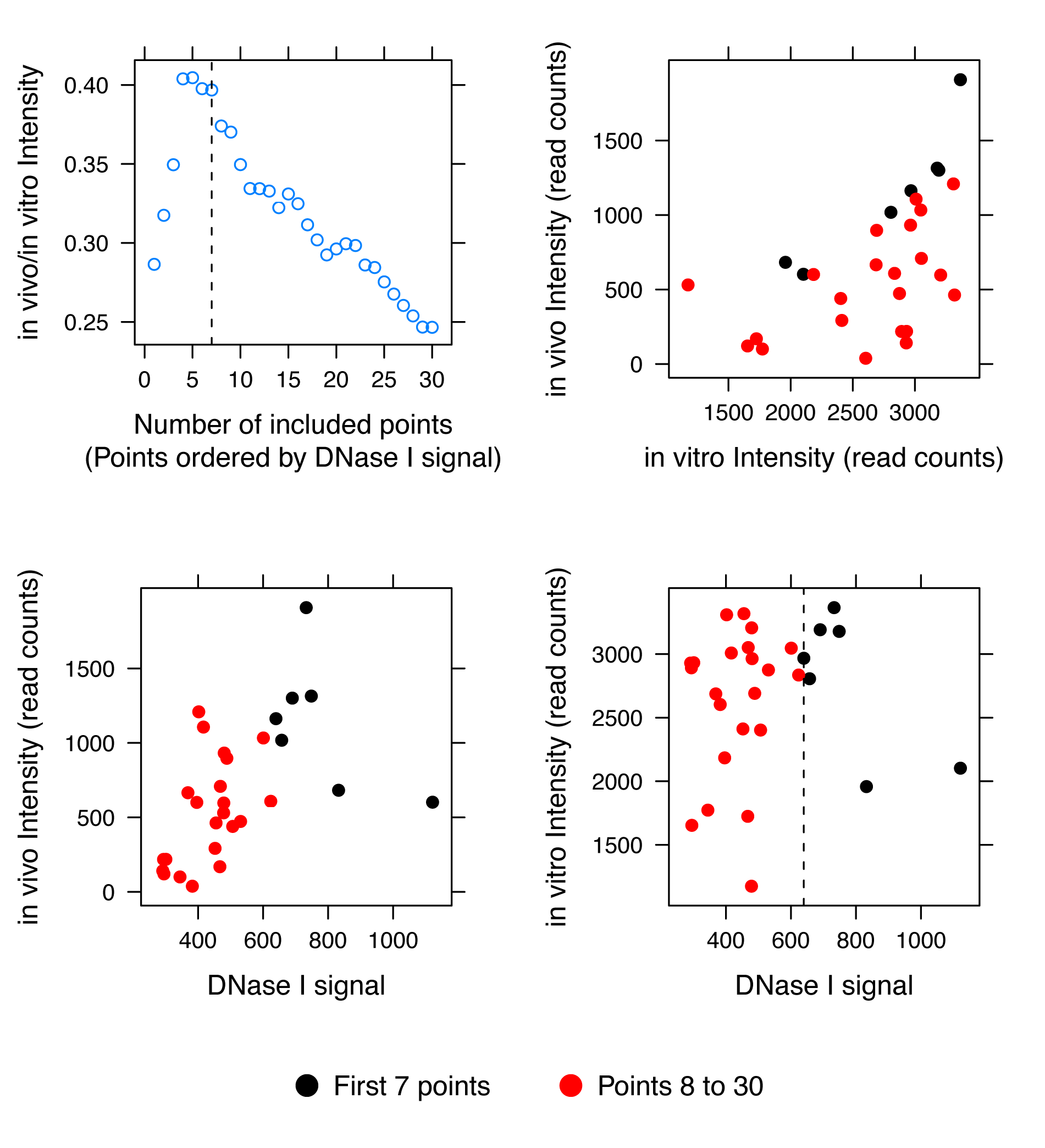

Supplement: Figure S4 — These data points (HSE cluster sites) were used to determine the scaling factor between in vivo and in vitro binding intensities in Figure 1 and Figure 3. The top left plot shows how the in vivo to in vitro intensity ratio varies with the number of points included; dashed line signals the final choice of seven points. Scatter plots show the top 30 data points (HSE cluster sites) with the highest DNase I signal, against their in vivo and in vitro intensity values; black indicates the seven chosen points. The points with higher DNase I hypersensitivity offer the best choice for unbiased scaling. (TIF) [file pgen.1002610.s004.tif]

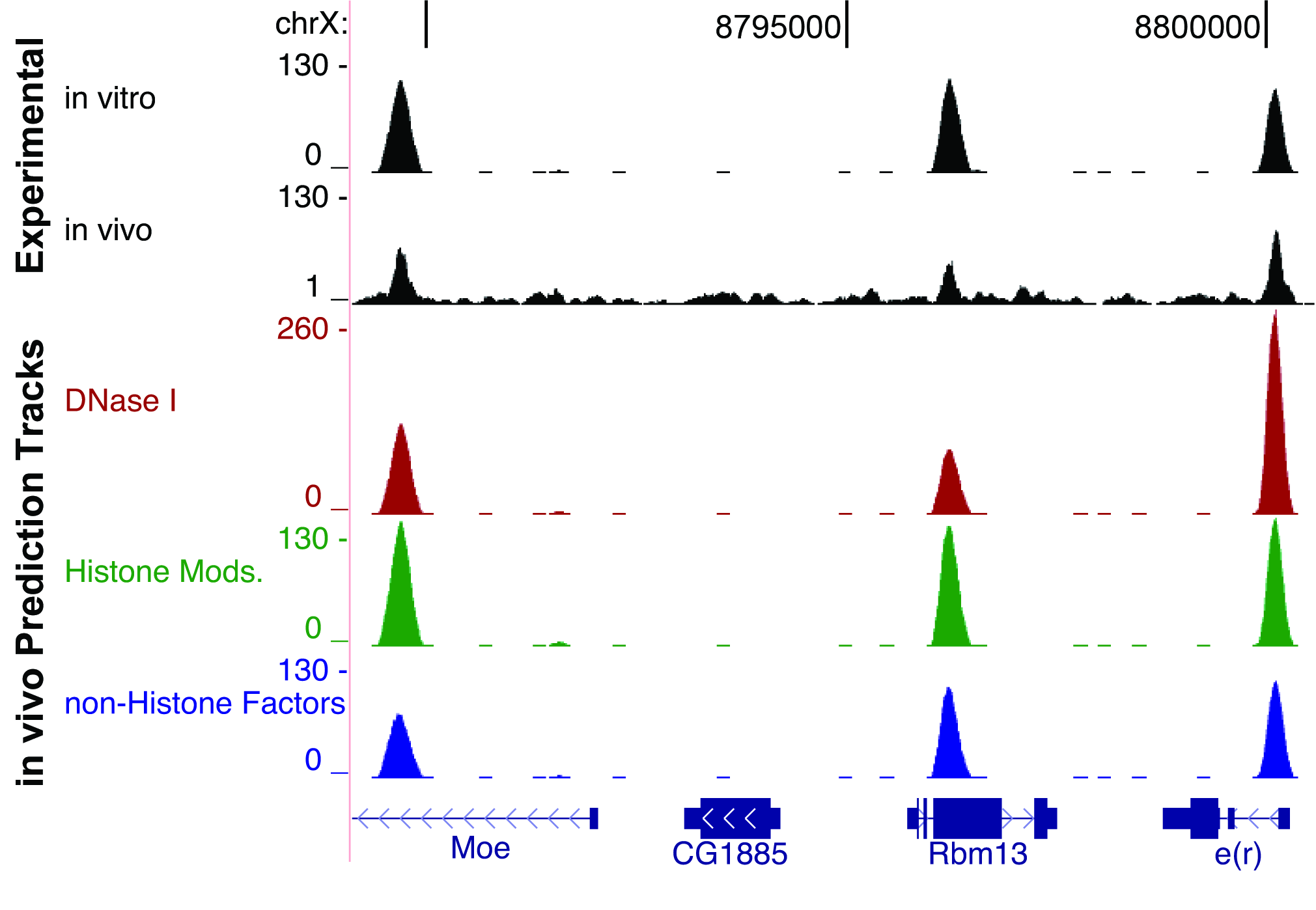

Supplement: Figure S5 — This UCSC genome browser shot provides additional examples of in vivo prediction of HSF binding intensity using chromatin and PB–seq data. (TIF) [file pgen.1002610.s005.tif]

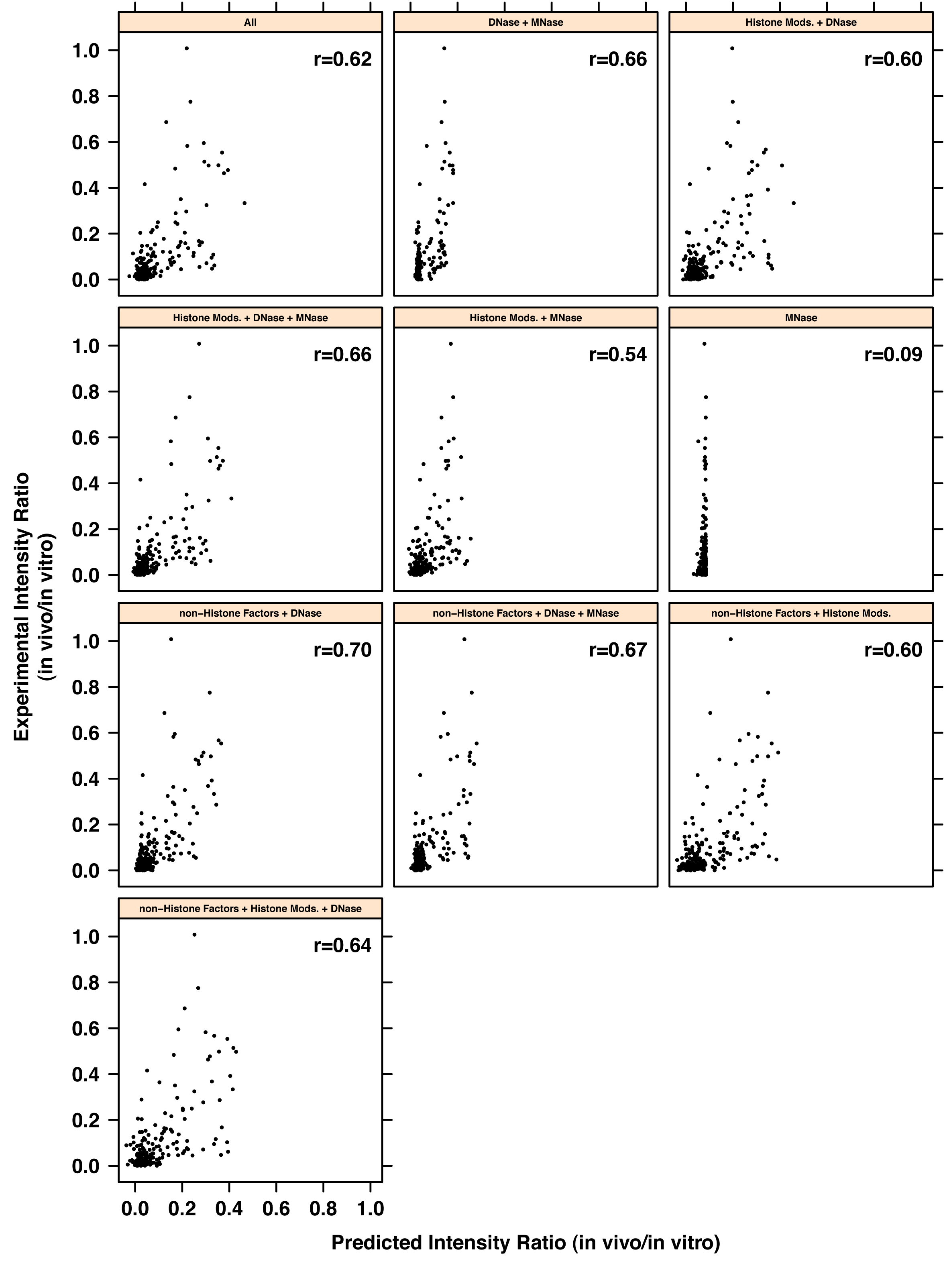

Supplement: Figure S6 — The experimentally determined ratio between in vivo ChIP-seq HSF intensity and in vitro PB–seq intensity is plotted against the predicted in vivo/actual PB–seq ratio. The Pearson correlation for each model is shown. (TIF) [file pgen.1002610.s006.tif]

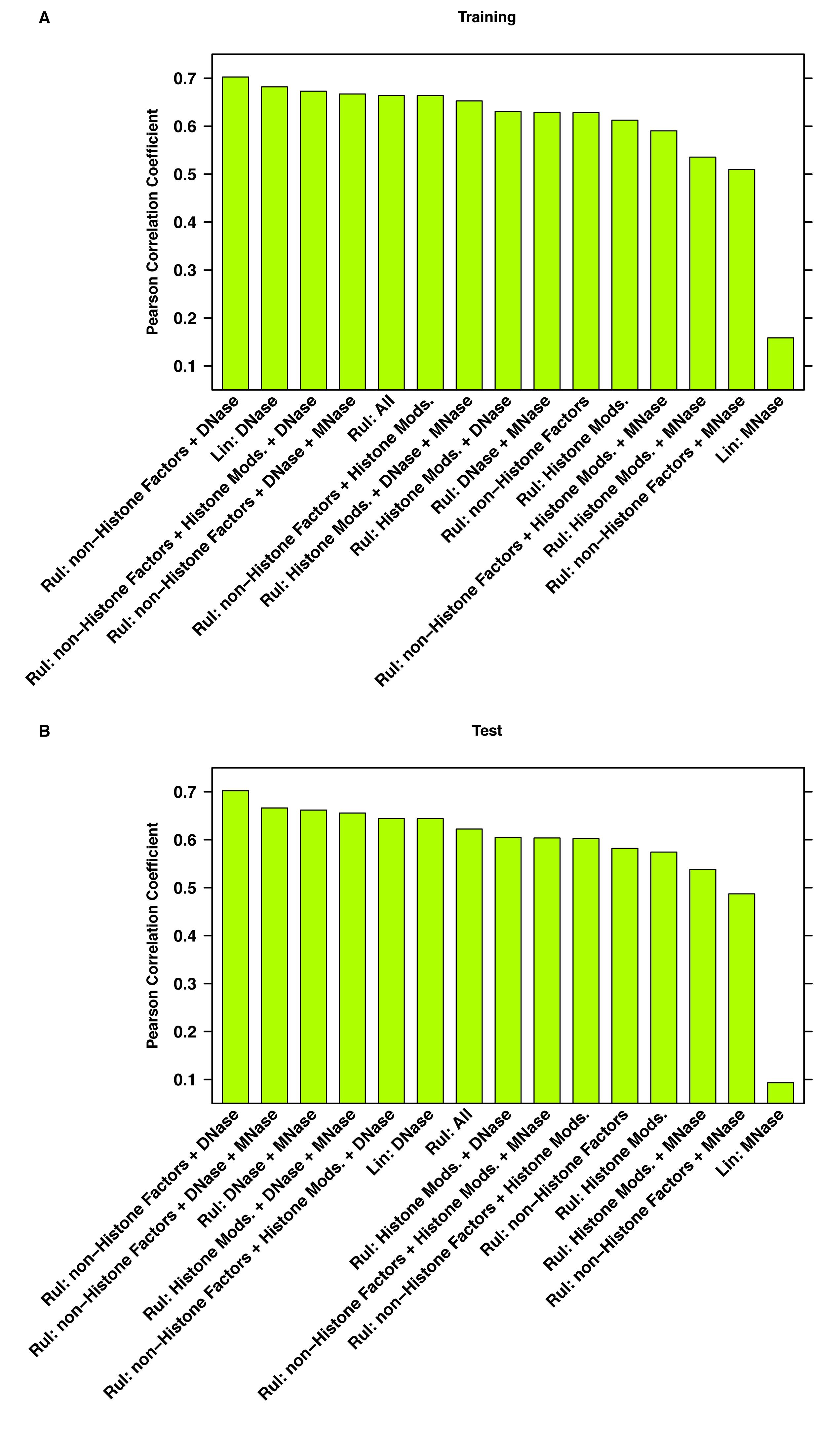

Supplement: Figure S7 — The bar graphs indicate the Pearson correlation of predictions versus experimental measures for each model used to predict the in vivo/in vitro binding intensity ratio (Rul: Rules Ensemble model, Lin: linear regression model). The correlations for both the training data (panel A) and the test data (panel B) are indicated. (TIF) [file pgen.1002610.s007.tif]

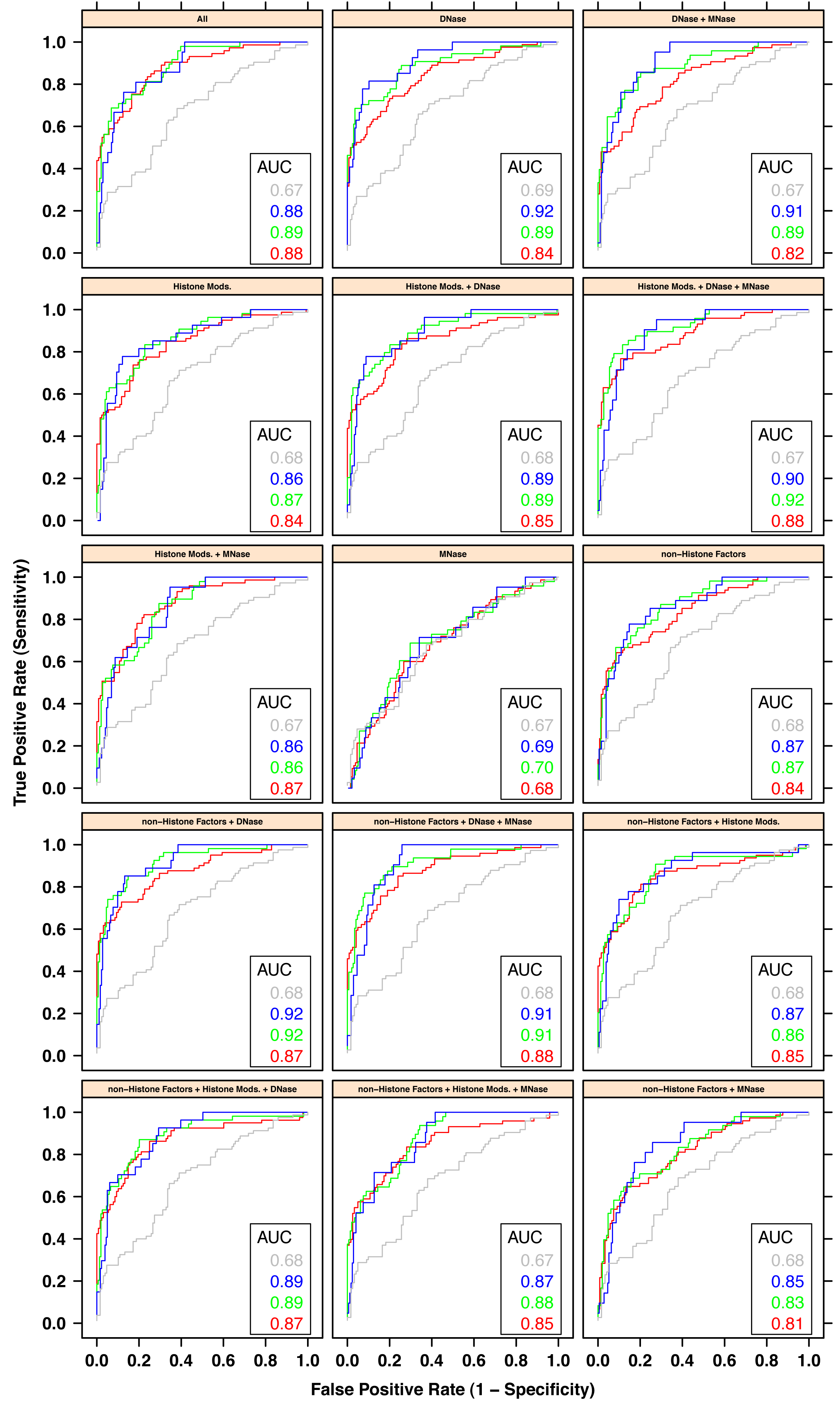

Supplement: Figure S8 — ROC plots for in vivo HSF binding predictions. In vitro HSE sites were partitioned into bound and unbound cases by applying a threshold to the estimated in vivo intensity values. Three thresholds were considered: a permissive threshold (shown in red; 36% bound), a moderate threshold (green; 24% bound) and a strict threshold (blue; 12% bound). Each panel in the figure represents a distinct covariate set (see panel titles). For each covariate set, the corresponding rules ensemble model was applied to predict the in vivo intensity of the HSE sites. Each site was then classified as predicted to be bound or unbound by applying a threshold to these predicted intensities. These thresholds were varied to produce the Receiver Operating Characteristic (ROC) curves shown. As a baseline, we show predictions based on the scaled in vitro intensities in gray. For each ROC curve, we compute the Area Under the Curve (AUC) as a general measure of prediction performance (higher is better). Notice that the ROC curves are not highly sensitive to the threshold that is applied to the in vivo intensities, but in most cases the ensemble model produces a substantial improvement over the baseline prediction. At the same time, some covariates produce substantially better predictions than others. (TIF) [file pgen.1002610.s008.tif]

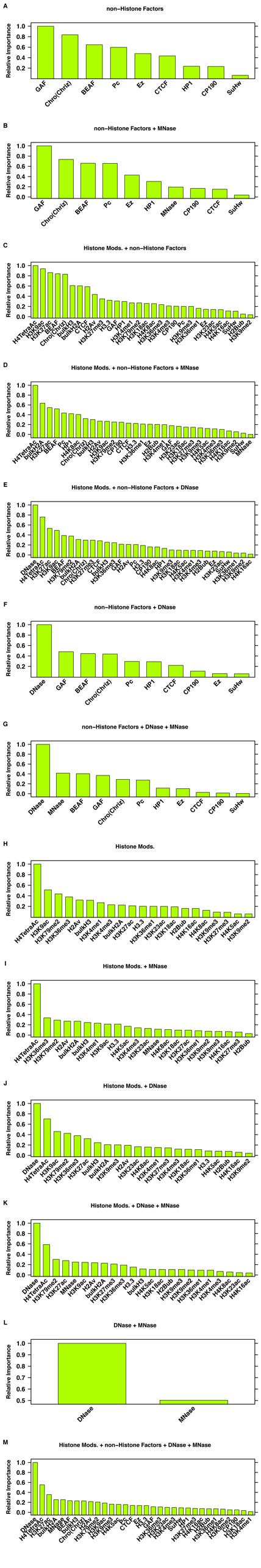

Supplement: Figure S9 — For each of the models shown in Figure S6 we show the relative importance [20] of each covariate in the Rule Ensemble model built with each indicated subset of covariates to predict the in vivo/in vitro binding intensity ratio. (TIF) [file pgen.1002610.s009.tif]

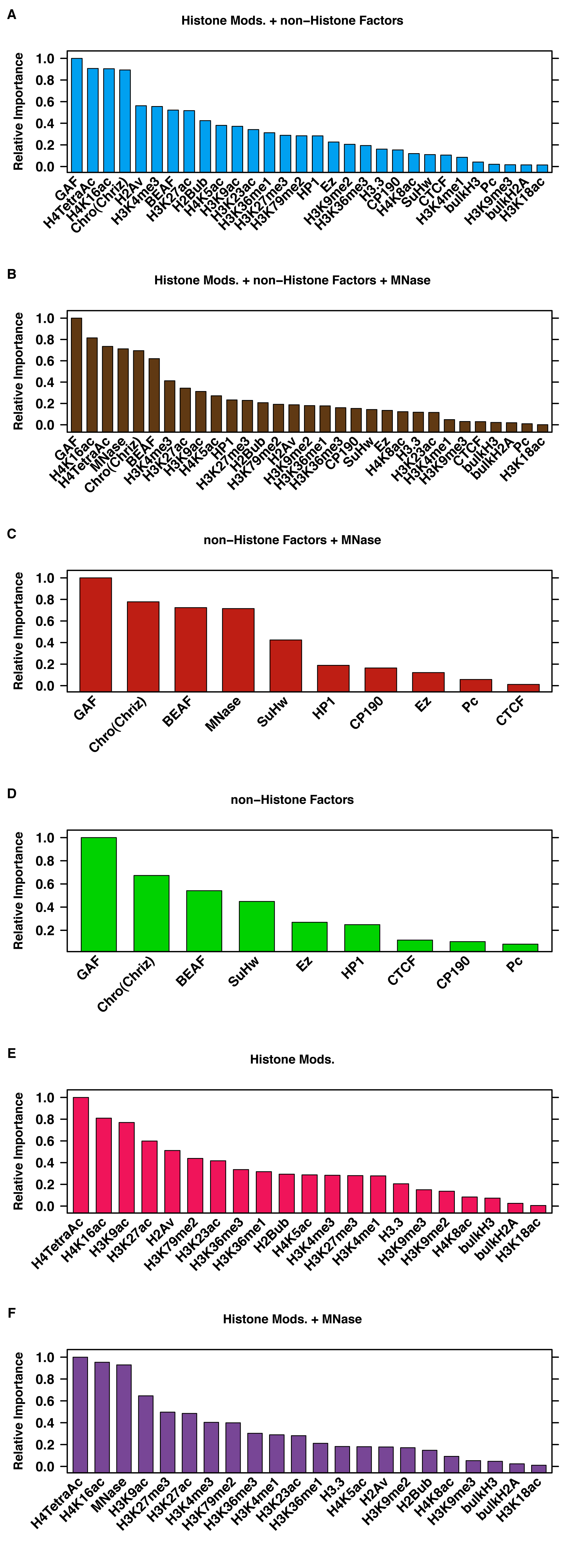

Supplement: Figure S10 — The bar graphs illustrate the relative importance [20] of each covariate in the Rule Ensemble model built with each indicated subset of covariates to predict DNase I hypersensitivity. (TIF) [file pgen.1002610.s010.tif]

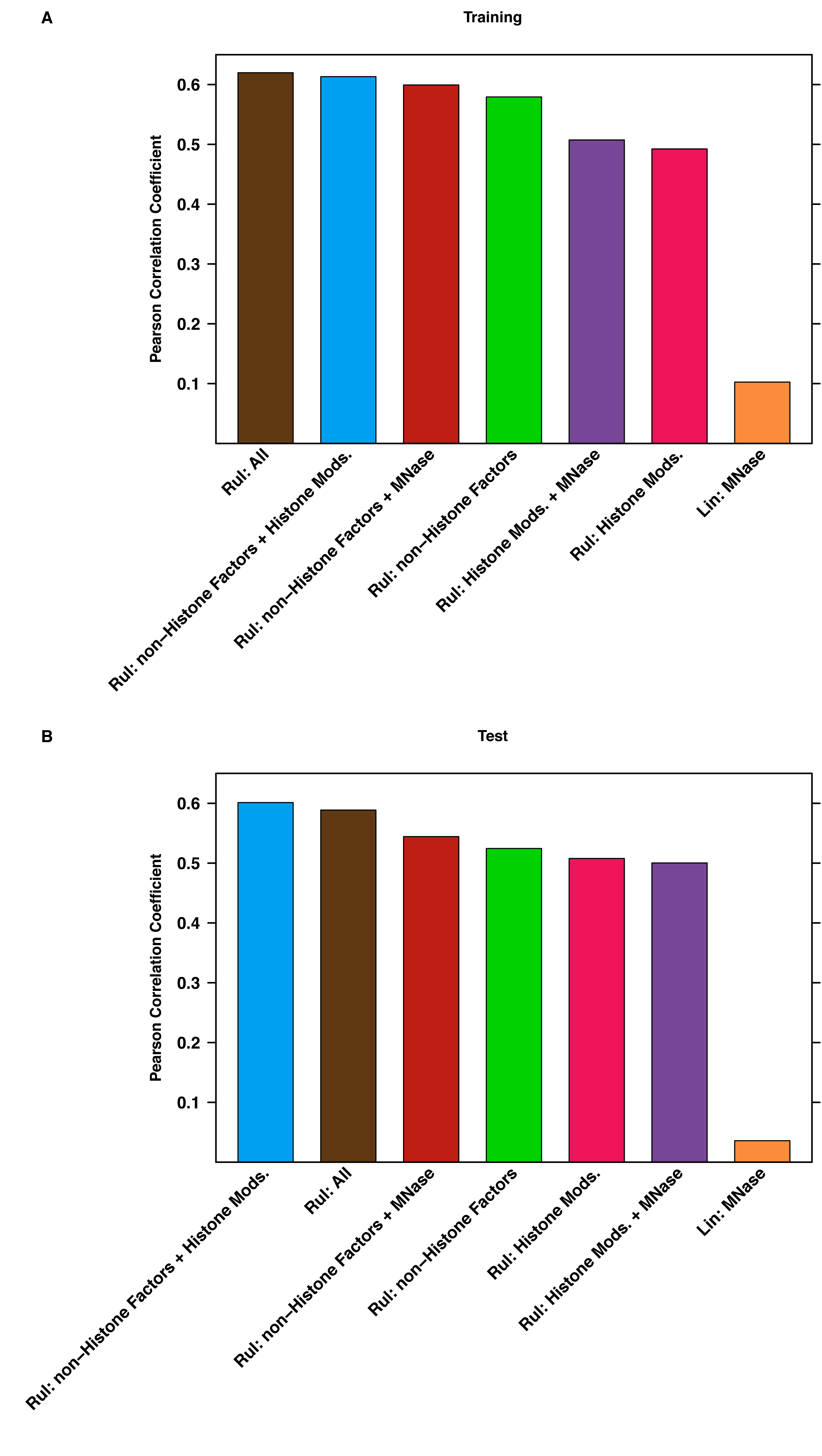

Supplement: Figure S11 — These bar graphs indicate the Pearson correlation of predicted versus experimentally measured DNase I sensitivity for each DNase I prediction model (Rul: Rules Ensemble model, Lin: linear regression model). The correlations for the training data (panel A) and test data (panel B) are indicated. (TIF) [file pgen.1002610.s011.tif]

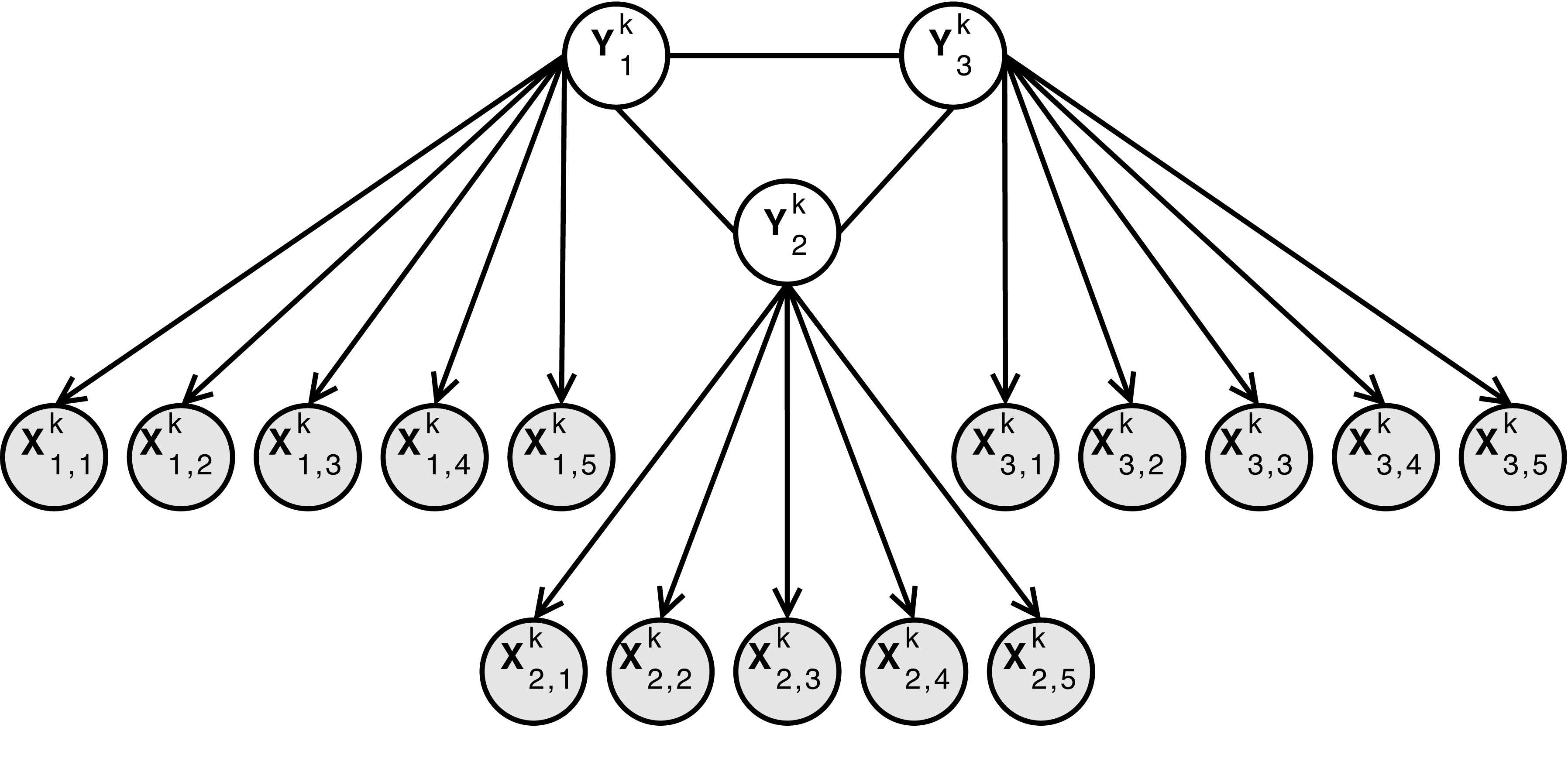

Supplement: Figure S12 — The structure of the HSE probabilistic sequence model recapitulates the structure of the HSE. Each hidden variable Y1,Y2,Y3, determines if the respective underlying pentamer bases are drawn from a strict base distribution or a relaxed version. (TIF) [file pgen.1002610.s012.tif]

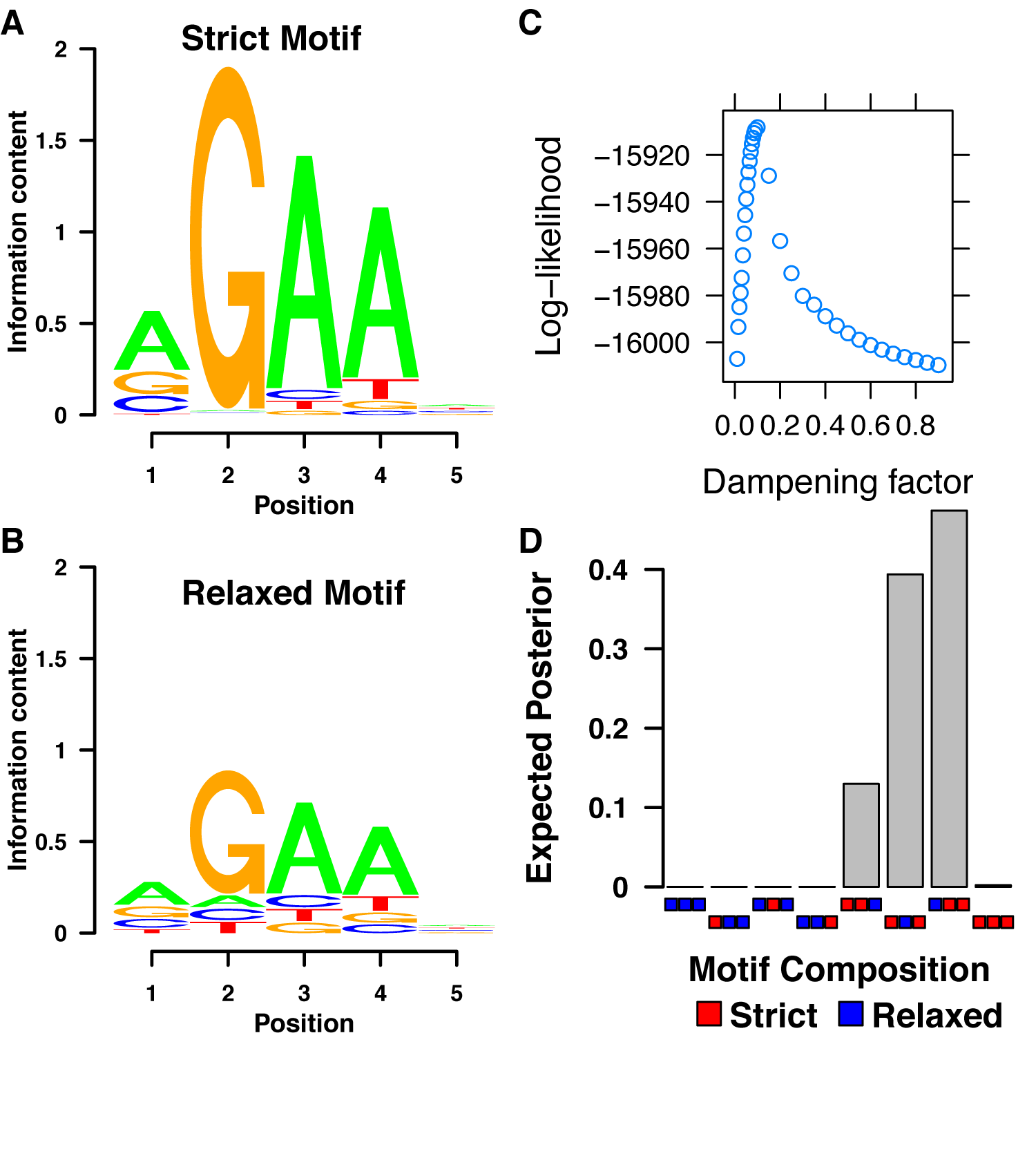

Supplement: Figure S13 — Pentamers within the HSEs are dependent upon their stringency and position relative to the other pentamers. A) A composite pentamer matrix was derived from all pentamers found within PB–seq peaks. B) The strict motif from panel A and a dampening factor from panel C were used to generate a relaxed motif. C) The dampening factor was optimized to generate a relaxed motif that best explained the data. D) A probabilistic sequence model reveals that the presence of two strict and one relaxed pentamer provides the best explanation of the data. (TIF) [file pgen.1002610.s013.tif]

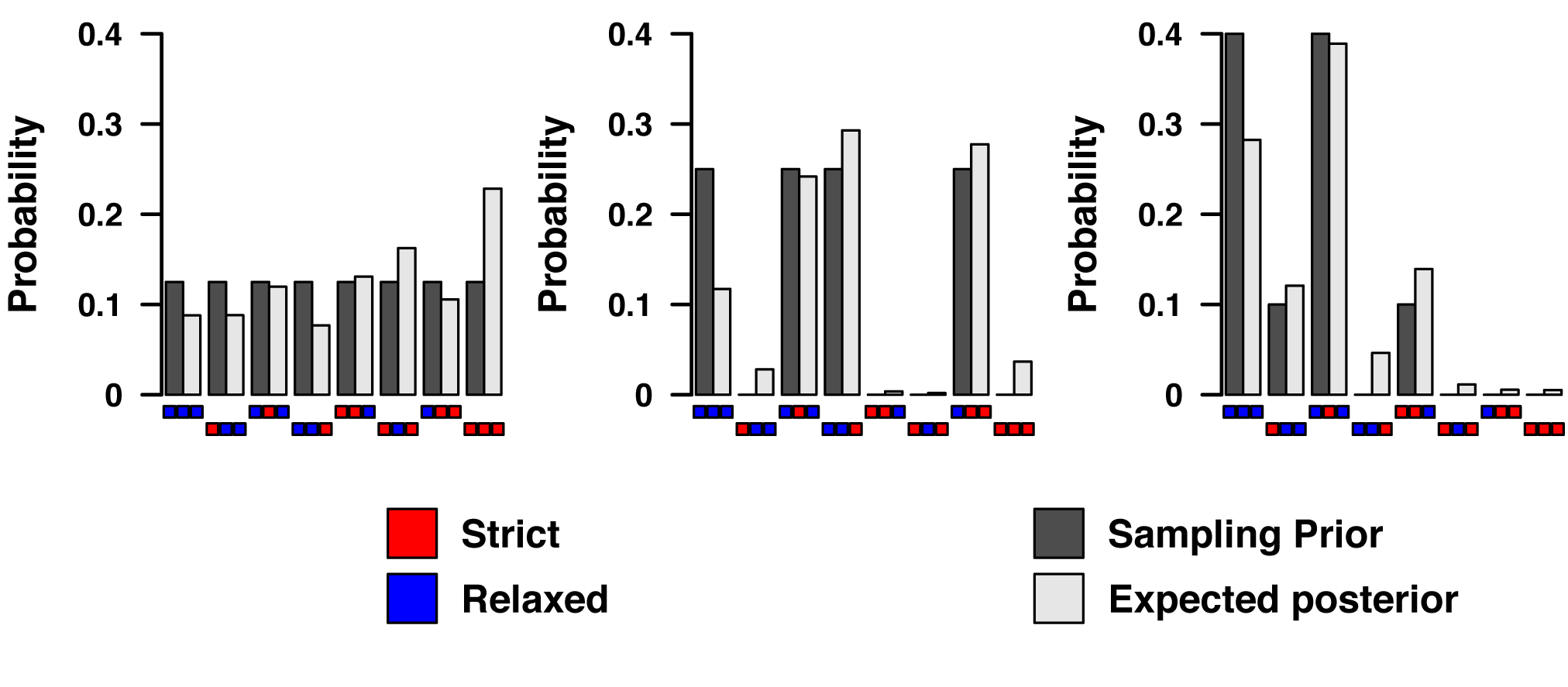

Supplement: Figure S14 — The reduced HSE sequence model predictions are compared for patterns of strict/relaxed pentamer combinations. Three different simulated patterns are shown and are recapitulated by the model. (TIF) [file pgen.1002610.s014.tif]

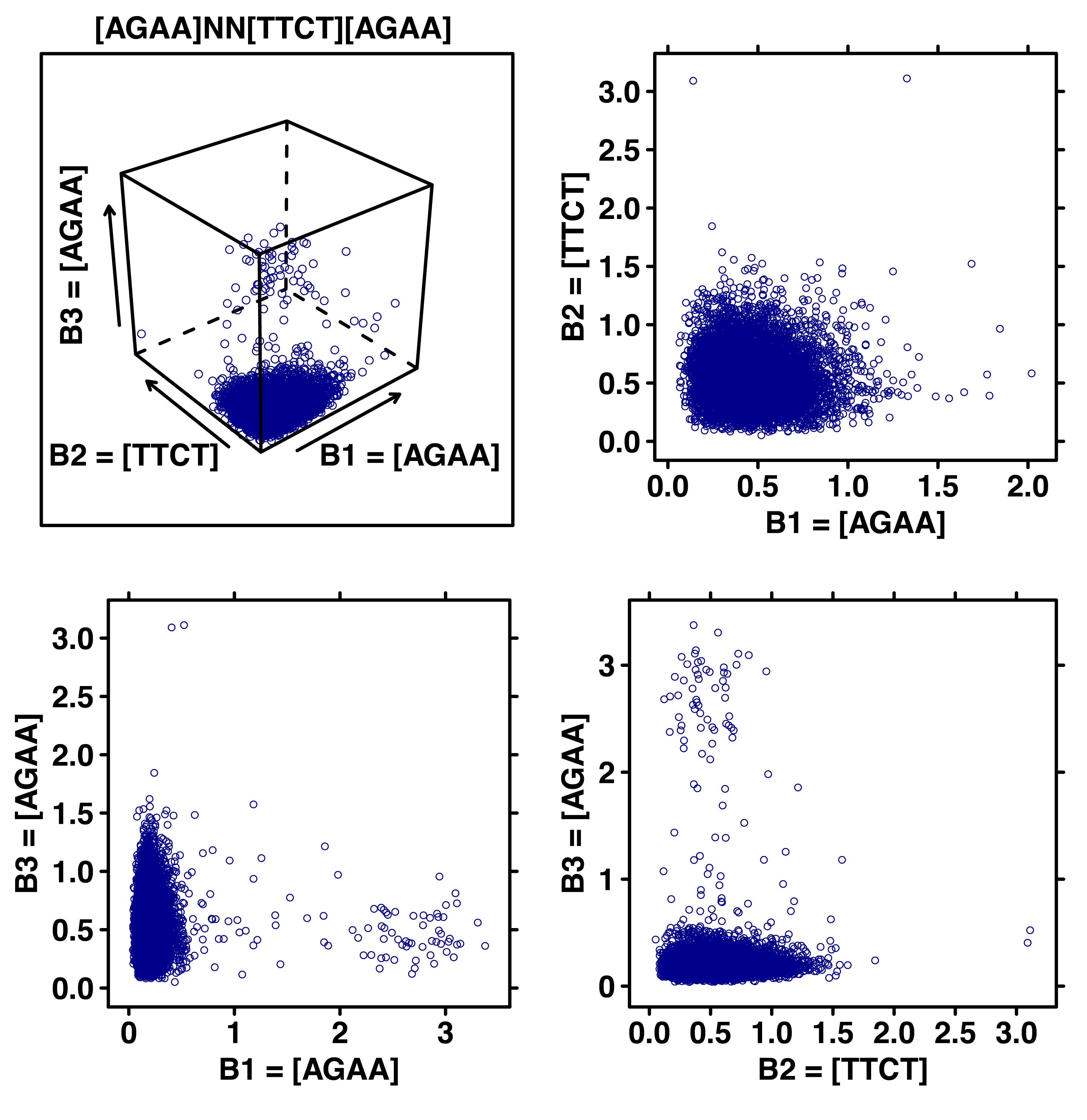

Supplement: Figure S15 — Scatter plots show similarity of each HSE pentamer to the canonical monomer PSSM. Each point represents a PSSM estimated via MEME by sub-sampling the in vitro peaks identified by MACS. Pattern of the scatter plot shows evidence for pentamer divergence occurring on one pentamer at a time (points are spread following the axis, mainly corresponding relaxed versions of the first and second pentamers). (TIF) [file pgen.1002610.s015.tif]

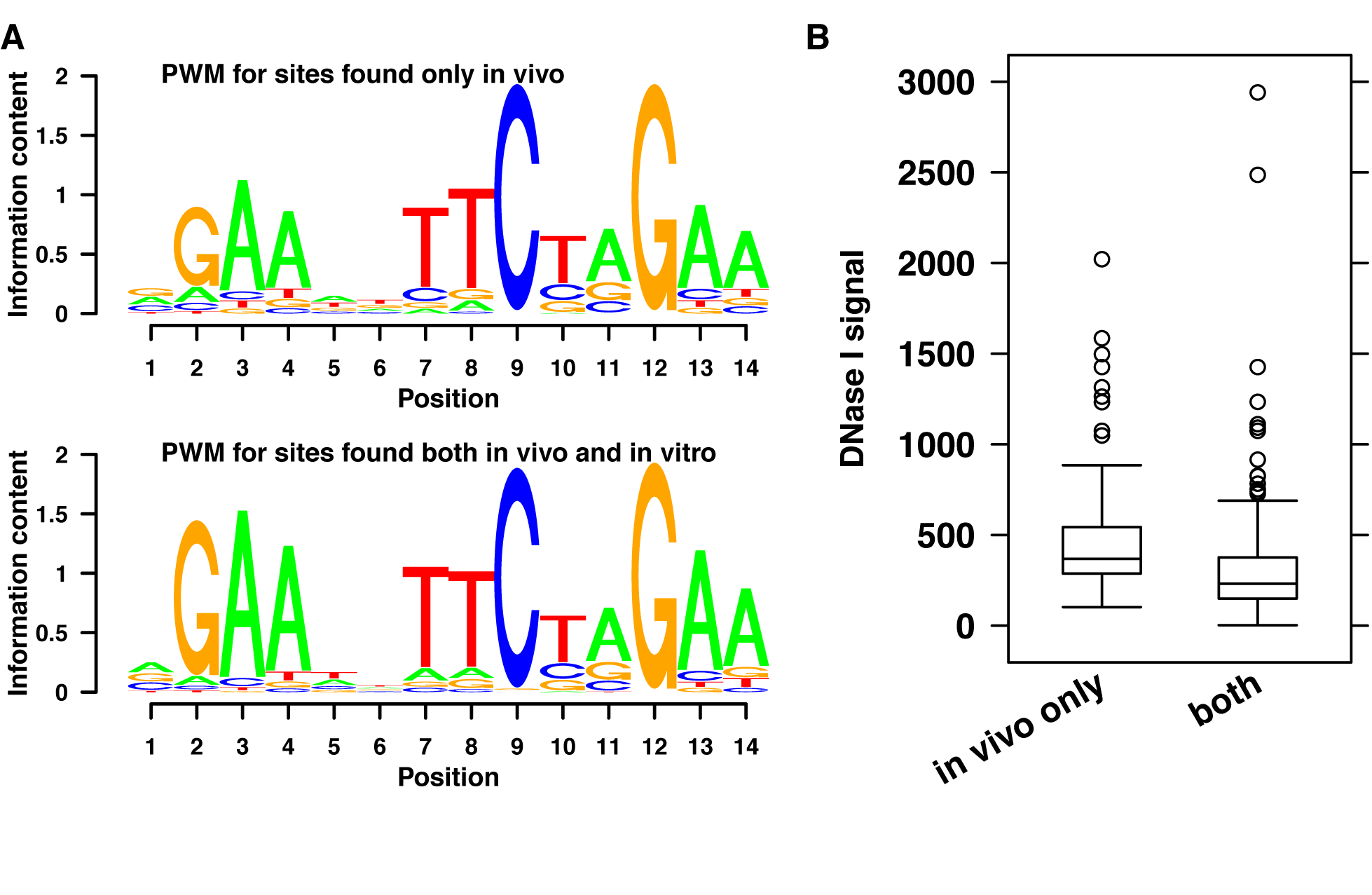

Supplement: Figure S16 — In vivo HSF binding sites that were either detected or not detected in vitro have distinct properties. A) The composite PSSM for the 40% of HSF binding sites that are only found in vivo exhibits more degeneracy than the PSSM from the sites that are found both in vivo and in vitro. B) The binding sites exclusively found in vivo are generally more accessible, as measured by DNase I signal, than those sites found both in vivo and in vitro. (TIF) [file pgen.1002610.s016.tif]

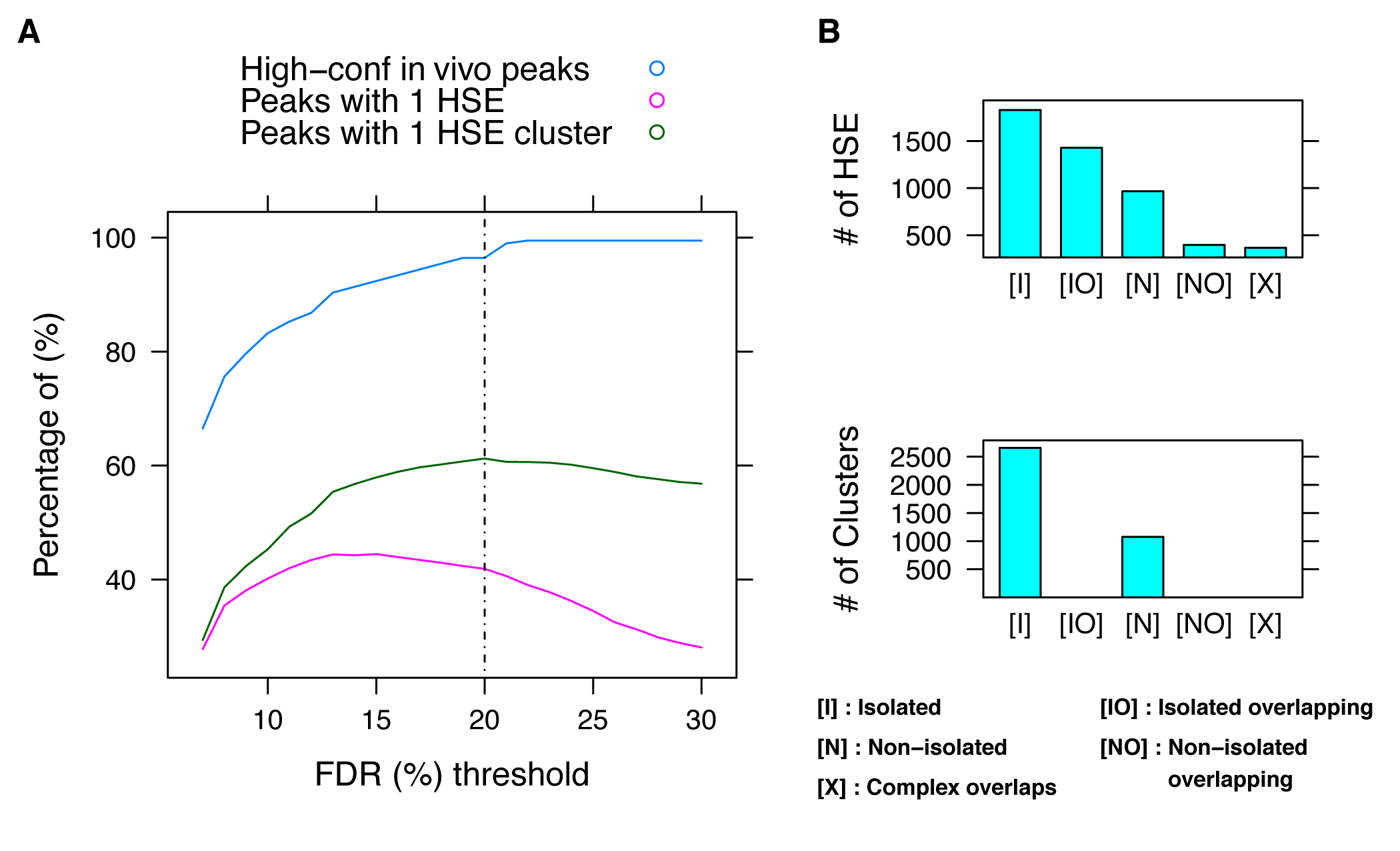

Supplement: Figure S17 — A) Balance between in vivo recall and number of per peak in vitro HSE is reached at 20% estimated FDR, corresponding to the inflection point for the number of clusters, as well as near maximal recall of high-confidence in vivo sites. B) An HSE (or HSE cluster) is considered isolated if the nearest neighbor is more than 200 bp away. An HSE (or HSE cluster) is considered overlapping if it overlaps with a single other HSE (or HSE cluster); overlaps between more than two HSE (or HSE clusters) are denoted as complex overlaps. (TIF) [file pgen.1002610.s017.tif]

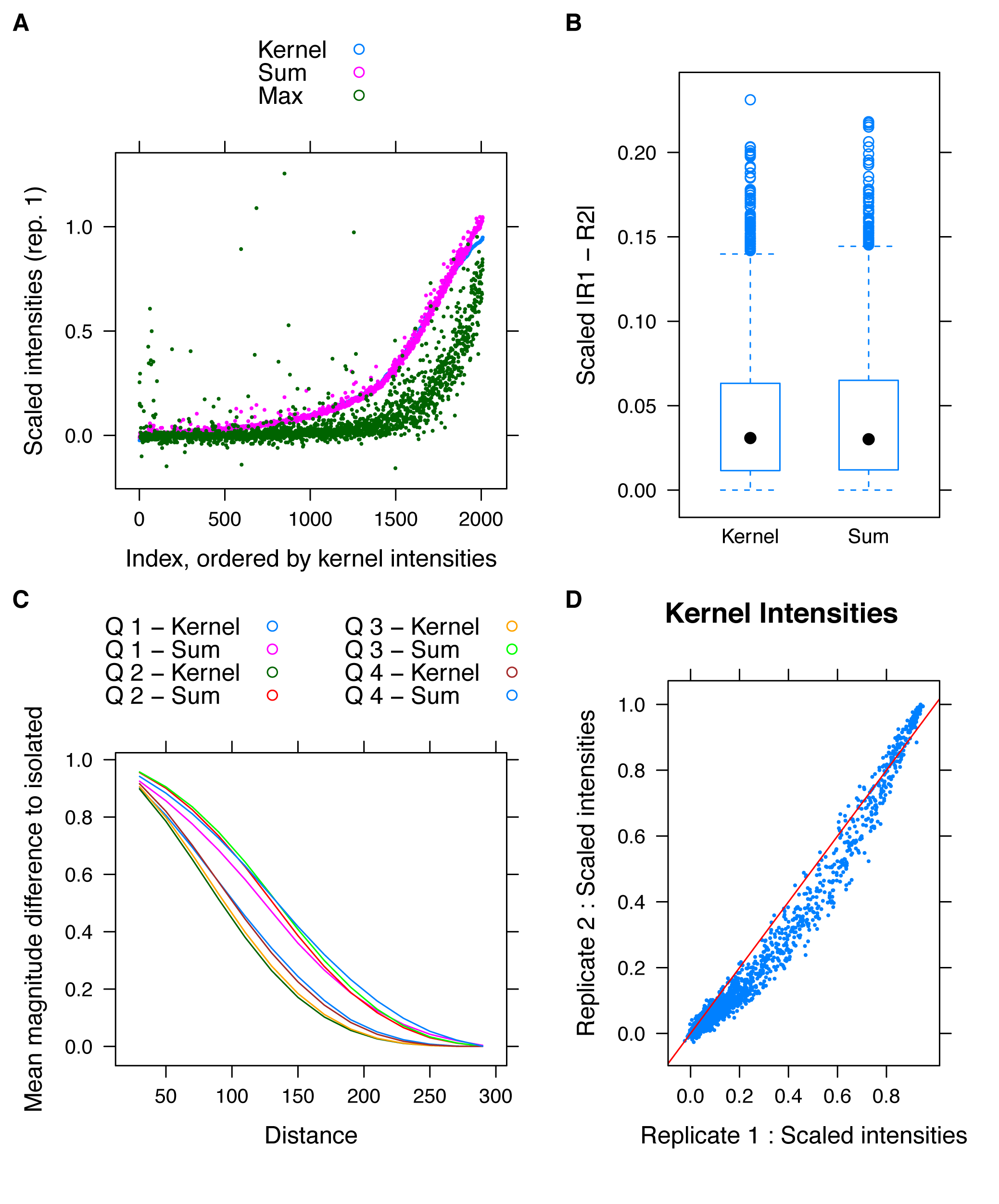

Supplement: Figure S18 — Three different measures were compared for computing HSE cluster intensities: max, sum and bi-weight kernel. A) Each measure was incorporated into a scatter plot of scaled intensities. Max was rejected because it produced a more compressed range of intensity values. B) In comparing the difference in intensities across replicates, the bi-weight kernel approach fares slightly better than the sum. C) The difference in magnitudes given a cluster distance on isolated clusters was compared between measures. For each distance, the isolated clusters are made to overlap an identical copy of themselves and the magnitude difference is computed by comparing the value of the isolated cluster with the partially overlapping, using either the sum or kernel measures. Average values per intensity quartile show that bi-weight kernel measure introduces less error as a function of distance than the sum measure. D) Replicate intensities strongly correlate, as predicted, using the kernel measure. (TIF) [file pgen.1002610.s018.tif]
